# Physiological and Biochemical Responses of *Lavandula angustifolia* to Salinity Under Mineral Foliar Application

**DOI:** 10.3389/fpls.2018.00489

**Published:** 2018-04-20

**Authors:** Antonios Chrysargyris, Evgenia Michailidi, Nikos Tzortzakis

**Affiliations:** Department of Agricultural Sciences, Biotechnology and Food Science, Cyprus University of Technology, Limassol, Cyprus

**Keywords:** antioxidants, essential oil, *Lavandula angustifolia*, salinity, cation application, soilless culture

## Abstract

Saline water has been proposed as a solution to partially cover plant water demands due to scarcity of irrigation water in hot arid areas. Lavender (*Lavandula angustifolia* Mill.) plants were grown hydroponically under salinity (0–25–50–100 mM NaCl). The overcome of salinity stress was examined by K, Zn, and Si foliar application for the plant physiological and biochemical characteristics. The present study indicated that high (100 mM NaCl) salinity decreased plant growth, content of phenolics and antioxidant status and essential oil (EO) yield, while low-moderate salinity levels maintained the volatile oil profile in lavender. The integrated foliar application of K and Zn lighten the presumable detrimental effects of salinity in terms of fresh biomass, antioxidant capacity, and EO yield. Moderate salinity stress along with balanced concentration of K though foliar application changed the primary metabolites pathways in favor of major volatile oil constituents biosynthesis and therefore lavender plant has the potential for cultivation under prevalent semi-saline conditions. Zn and Si application, had lesser effects on the content of EO constituents, even though altered salinity induced changings. Our results have demonstrated that lavender growth/development and EO production may be affected by saline levels, whereas mechanisms for alteration of induced stress are of great significance considering the importance of the oil composition, as well.

## Introduction

Salinity is the condition in soil characterized by a high concentration of soluble salts and is one of the major factors that affects plant growth, causing considerable losses in agricultural production (Wu et al., 2007). The harmful effects of salinity on plant growth are related with (a) low osmotic potential of soil solution (water stress), (b) nutritional imbalance (synergism-antagonism), (c) specific ion effect (salt stress), or (d) a mixture of these factors (Yildirim and Taylor, 2005). Saline soils are generally dominated by sodium, chloride and sulfate ions, with high sodium absorption rate and with high pH and electrical conductivity (EC > 4.0 dS/m) (Flowers and Flowers, 2005).

The high saline condition determines high osmotic pressure in the rhizosphere and eventually reduces plant water and nutrients availability, which in turn affect crops' primary and secondary metabolism (Hendawy and Khalid, 2005). Plants utilize molecular O_2_ as a terminal electron acceptor. For this reason, reactive oxygen species (ROS), such as singlet oxygen (O_2_^1^), superoxide (${\text{O}}_{2}^{-}$), and hydrogen peroxide (H_2_O_2_) are normally produced by metabolism in all cellular compartments. In particular, the electron transport chain is responsible for most of the superoxide produced through partial reduction of oxygen (Bolisetty and Jaimes, 2013). They can destroy normal metabolism through oxidative damage of lipids, proteins, and nucleic acids when they are produced in excess as a result of oxidative stress (Gill and Tuteja, 2010). In order to overcome oxidative-related stress, together with non-enzymatic antioxidant molecules (ascorbate, glutathione, α-tocopherol etc.), plants detoxify ROS by up-regulating antioxidative enzymes like superoxide dismutase (SOD), catalase (CAT), peroxidase (POX), glutathione peroxidase (GPX), glutathione *S*-transferases (GST), ascorbate peroxidase (APX), dehydroascorbate reductase (DHAR), glutathione reductase (GR), and monodehydroascorbate reductase (MDHAR) (Turkan and Demiral, 2009). SOD provides the first line of defense against the toxic effects of ROS-elevated levels. The SODs convert ${\text{O}}_{2}^{-.}$ to H_2_O_2_ while H_2_O_2_ is a strong nucleophilic oxidizing agent and the oxidation of SH-group is one of the major mode of its toxicity. The produced H_2_O_2_ is then scavenged by catalase and a variety of peroxidases (Tarchoune et al., 2010). Catalase dismutates H_2_O_2_ into water and molecular O_2_, whereas POX decomposes H_2_O_2_ by oxidation of co-substrates such as phenolic compounds and/or antioxidants. In plant cells the ascorbate/glutathione (ASH-GSH) cycle represents an alternative and more efficient detoxification mechanism against H_2_O_2_, operating both in the chloroplasts and the cytosol (Sgherri and Navari-Izzo, 1995). It may remove H_2_O_2_ in a series of enzymatic reactions involving APX and GR (Ahmad et al., 2010; Gill and Tuteja, 2010). Therefore, the two likely harmful superoxide and hydrogen peroxide are converted to water.

In saline stress condition, both SOD and CAT activities are reduced and malondialdehyde (MDA) accumulates rapidly in plants (Fadzilla et al., 1997; Woodrow et al., 2017), resulting in higher plasma membranes permeability. Additionally, the production of antioxidant compounds serves as a prominent defense trait under environmental stress (de Abreu and Mazzafera, 2005). Actually the biosynthesis of polyphenols may be accelerated in response to abiotic constraints (Naczk and Shahidi, 2004) and saline-stressed plants might represent a promising source of polyphenols (Taarit et al., 2012).

Sodium (Na) excess in roots disrupt plant nutrition, especially potassium (K) absorption. Potassium deficiency inevitably reduce plant growth as it plays a critical role in maintaining cell turgor, membrane potential and enzyme activity (Jouyban, 2012). Once Na gets into the cytoplasm, it inhibits several enzymes activity, whereas a high Na/K ratio cause more damage (Jouyban, 2012). Potassium affects plant physiological processes such as photosynthesis, proteins metabolism, phloem transport, enzymes activity, and osmotic potential maintenance (Chérel, 2004). The monovalent cations, such as K, play a role in enzyme activation lowering energy barriers in the ground and/or transition states rather than being the agents themselves of causing catalysis (Page and Di Cera, 2006). In addition, K increases the vegetation and essential oils (EO) yield in numerous aromatic crops (Singh et al., 2007; Said-Al Ahl et al., 2009) and EO constituents (Hussien, 1995).

Zinc (Zn) is an essential component for over 300 enzymes in plants and makes up an integral constituent of the enzyme structure (Hendawy and Khalid, 2005). It is involved in the auxin synthesis, photosynthesis, cell division, membrane structure, and function maintenance, sexual fertilization and is thus linked to photosynthesis and carbohydrate metabolism (Said-Al Ahl and Omer, 2009; Marschner, 2012). Parker et al. (1992) suggested that root cell membrane permeability is increased under zinc deficiency which might be associated to the zinc functions in cell membranes. Alpaslan et al. (1999) reported that the application of zinc could alleviate possible Na and Cl injury in salt-stressed plants by preventing Na and/or Cl uptake or translocation. Foliar spraying (micro- and macronutrients) under saline conditions could be much more efficient than any other application of nutrients to the soil (El-Fouly et al., 2001). Several reports are reported to the zinc application in plants highlighting its stimulatory effects, such as on basil (Said-Al Ahl and Mahmoud, 2010), geranium (Misra et al., 2005), and coriander (Said-Al Ahl and Omer, 2009). Misra et al. (2005) stated that EO biosynthesis in geranium was greatly influenced by zinc acquisition or deficiency.

Silicon (Si) was disclosed to reduce the hazardous effects of various biotic and abiotic stresses counting salt and drought stress, metal toxicity, radiation damage, several pests, and diseases caused by both fungi and bacteria, nutrients imbalance, high, and low temperature-freezing (Ma, 2004). Silicon reduces the transpiration rate by suppressing the salt translocation from the rhizosphere to the shoot and thereby Si alleviates salt stress (Matlou, 2006). Si application increases phosphorus, calcium, and magnesium uptake but decreases the uptake of nitrogen and potassium (Liang et al., 2007). Salinity tolerance in plants by Si application include mechanisms related to the plant water status increase (Romero-Aranda et al., 2006), ROS stimulation (Zhu et al., 2004), toxic Na^+^ ion immobilization (Liang et al., 2003), and higher K^+^:Na^+^ selectivity by reduced Na^+^ uptake in plants (Hasegawa et al., 2000). In order to alleviate salinity induced stress, several protective (priming, develop salt-tolerant crops through breeding) and curative (cation and anion enrichment, plant growth-promoting rhizobacteria, controlled irrigation schedule, cultivation system) means have been examined (Gill and Tuteja, 2010; Tzortzakis, 2010; Chondraki et al., 2012; Ibrahim, 2016).

Nowadays, a great interest of Medicinal and Aromatic Plants exploitation is taking place due to their high antioxidant and antimicrobial activity, which surpassed many commonly used natural and synthetic antioxidants. Lavender is cultivated either as ornamental crop or aromatic crop for EO production and pharmaceutical uses. Therefore, lavender is used widely in medicines, balms, salves, perfumes, cosmetics and constitute a model plant for isoprenoid studies (Biswas et al., 2009). Several studies examined the effects of abiotic stress on lavender plants, including salinity (Cordovilla et al., 2014; Garcia-Caparros et al., 2017), water (Chrysargyris et al., 2016a), and mineral (Santos et al., 2016) stress.

Medicinal and Aromatic Plants properties are related to several vitamins, carotenoids, chlorophylls, catechins, phytoestrogens, minerals, etc. and contribute with plants and/or their antioxidant components for food preservation (Parejo et al., 2002). Recently, we have optimized the nutrition levels in lavender grown hydroponically, despite the less expansion of lavender intensive cultivation in soilless culture, in order to achieve a constant and repeatable EO composition during crop production (Chrysargyris et al., 2016b, 2017a). Moreover, little is known about salinity interaction with potassium, zinc and silicon deprivation. The objective of the present study was to examine the effects of saline levels and means for salinity induced stress overcome by mineral foliar application, when lavender is cultivated as (a) ornamental crop, (b) crop for essential oil production, or (c) crop for the leaves antioxidant quality. Therefore, it was studied several growth parameters, nutrient content, antioxidant activity as well as quality and quantity of essential oil of *Lavandula angustifolia* plant, just before flowering, as quite often plants are early harvested in an intermediate vegetative and flowering stage.

## Materials and methods

### Plant and experimental conditions

The experiment was carried out at the Hydroponic Infrastructures (non-heated fully controlled plastic greenhouse) of the Experimental Farm, Cyprus University of Technology, Limassol, Cyprus, during spring-summer (15 of April−29 of June) season of 2014. Average minimum and maximum air temperatures were 18 and 28°C respectively (Figure S1), as temperature reached up to 32–35°C during sunny hours in early summer.

Lavender (*L. angustifolia* Mill.) cuttings were purchased from the Cypriot National Centre of Aromatic Plants in trays at the stage of 3–4 leaves and 4–5 cm height. Cuttings were transplanted into pots (1 plant/pot) with perlite (5 L/pot). Pots arranged in twin rows (twin rows were 0.5 m apart and plants were separated by 0.2 m).

### Experimental design and treatments

Once lavender plants were adapted to soilless culture conditions, they were exposed for up to 60 days to four saline treatments and four foliar mineral applications. Each salinity treatment was divided into three complete randomized blocks, fertigated from the same nutrient solution tank of 180 L capacity. Each treatment consisted of six biological replications (three plants in each replication; 18 plants in total for each treatment) and were considered as experimental and measured further. The different nutrient solutions were applied to the plants considering four salinity levels (0–25–50 mM and 100 mM NaCl) with individual foliar spraying with dH_2_O, K (1,250 mg/L) using K_2_O, Zn (144 mg/L) using ZnSO_4_, and Si (725 mg/L) using SiO_2_ resulted in 16 treatments (Supplementary Image 1). The concentration used for foliar sprays were based on preliminary studies and/or previous reports (Tzortzakis, 2010). Foliar spays took place once every 2 weeks.

The soilless culture system was open or run-to-waste system, i.e., the drainage solution was thrown out of the system after fertigation and drainage. Nutrient solution used was based on previous findings (Chrysargyris et al., 2016b, 2017a). A solution (1:100 v/v) in water containing the following concentration of nutrients was used: ${\text{NO}}_{3}^{-}$-N = 14.29, K = 8.31, PO_4_-P = 2.26, Ca = 7.48, Mg = 5.76, ${\text{SO}}_{4}^{-2}$-S = 1.56, and Na = 1.91 mmol/L, respectively; and B = 18.21, Fe = 71.56, Mn = 18.21, Cu = 4.72, Zn = 1.53, and Mo = 0.52 μmol/L, respectively. Fertigation was applied during daytime through a timer (eight times with 1 min every time at a flow rate of 30 mL/min, due to low water holding capacity of perlite medium) with a drip irrigation system (via emitters; one emitter/plant) by means of pressure pumps (Einhell BG-GP 636, Germany). Fertigation was adjusted appropriately according to the plant needs and climatic conditions as fertigation management has been described previously (Chrysargyris et al., 2016b, 2017a). Pots were placed on swallow plates in order to collect partly the drainage solution and therefore, drainage solution was available to roots through capillary suction. Periodically, plates were washed out to eliminate any salt accumulation. The pH of the nutrient solution was set to 5.8 for all treatments and adjusted with diluted nitric acid (5% v/v). The standard EC of the nutrient solution was 2.1 mS/cm for the control treatment (0 mM NaCl), 5.0 mS/cm for the 25 mM NaCl, 9.0 mS/cm for the 50 mM NaCl and 14.0 mS/cm for the 100 mM NaCl.

### Measurement of growth parameters

Plant height, leaf length, stem thickness, shoot number, root length, plant fresh and dry weight for upper and root part were observed after 60 days of plant growth in six plants from each treatment. For measurement of fresh and dry weights of upper biomass (leaves and stems) and roots, respective plant parts were excised from control, NaCl-treated and foliar-treated plants and the fresh weight was noted immediately. Later, these plant parts were wrapped in pre-weighed aluminum foils and kept in an incubator at 75°C for 72 h before the dry weight was recorded.

### Measurement of physiological parameters

Stomatal conductance in six leaves (2nd–3rd from the top, fully mature sun-exposed leaf) in different individual plants per treatment was measured using a ΔT-Porometer AP4 (Delta-T Devices-Cambridge, UK) according to the manufacturer's instructions at the end of the experiment. For leaf chlorophyll determination, lavender leaf tissues (six replications/treatment; each replication was pooled out of two individual plants; leaf: 0.1 g) were incubated in heat bath at 65°C for 30 min, in the dark, with 10 mL dimethyl sulfoxide (DMSO, Sigma Aldrich, Germany) for chlorophyll extraction. The extract absorbance was measured at 645 and 663 nm (TECAN, infinite M200PRO, Männedorf, Austria). Photosynthetic leaf pigments, chlorophyll a (Chl a), chlorophyll b (Chl b), and total chlorophyll (t-Chl) concentrations were calculated (Chrysargyris et al., 2016b).

### Estimation of plant mineral ion contents

Leaf (six replications/treatment) and root (three replications/treatment) plant tissue samples were dried at 75°C for 4 d, weighted, and grounded in a Wiley mill to pass through a 40 mesh screens. Sub samples (0.2–0.3 g) were acid digested (2 N HCl). Determination of K, P, Ca, Mg, Fe, Cu, Mn, Zn, Na, and B was done by inductively coupled plasma optical spectrometry [ICP-OES; PSFO 2.0] (Leeman Labs INC., Hudson, USA) and N by the Kjeldahl (BUCHI, Digest automat K-439 and Distillation Kjelflex K-360) method.

### Essential oil extraction and analysis

Lavender plant harvested and three biological samples (pooled of three individual plants/sample) for each treatment were air-dried at 42°C in oven, chopped and were hydrodistilled for 3 h, using Clevenger apparatus for EO extraction. The EO yield was measured (%) and oils were analyzed by Gas Chromatography-Mass Spectrometry (GC/MS- Shimadzu GC2010 gas chromatograph interfaced Shimadzu GC/MS QP2010plus mass spectrometer) and constituents were determined (Chrysargyris et al., 2016b).

### Polyphenol content and antioxidant activity of lavender

Polyphenols were extracted from six samples (two individual plants were pooled/sample) for each treatment. Plant tissue (0.5 g) was milled (for 60 s) with 10 mL methanol (50% v/v) and extraction was assisted with ultrasound for 30 min. The samples were centrifuged for 15 min at 4,000 g at 4°C (Sigma 3–18 K, Sigma Laboratory Centrifuge, Germany). Extracts were stored at −20°C until use for analysis of total phenolic and total antioxidant activity by the 2,2-diphenyl-1-picrylhydrazyl (DPPH), ferric reducing antioxidant power (FRAP) and 2,2′-Azino-bis-3-ethylbenzothiazoline-6-sulfonic acid (ABTS) method.

Total phenols content was determined using Folin-Ciocalteu method at 755 nm according to Tzortzakis et al. (2011) and results were expressed as equivalents of gallic acid (Scharlau, Barcelona, Spain) per g of fresh weight (mg of GAE/g Fwt). DPPH and FRAP radical-scavenging activity was determined as described previously (Chrysargyris et al., 2016b). In details, DPPH radical scavenging activity of the plant extracts was measured at 517 nm from the bleaching of the purple-colored 0.3 mM solution of DPPH. Standard curve was prepared using different concentrations of trolox [(±)-6-hydroxy-2,5,7,8-tetramethylchromane-2-carboxylic acid], and results were expressed as mg trolox/g Fwt. FRAP radical scavenging activity of the plant extracts was measured at 593 nm and the results were expressed as mg trolox/g Fwt (Chrysargyris et al., 2016b). The antioxidant capacity using the ABTS method was carried out according to Wojdylo et al. (2007) and results were expressed as mg trolox/g Fwt.

### Damage index: determination of content of H_2_O_2_ and lipid peroxidation

The content of H_2_O_2_ was determined according to Chrysargyris et al. (2017b), from six samples (two individual plants were pooled/sample) for each treatment. Leaf tissue (0.2 g) was ground in ice cold 0.1% trichloroacetic acid (TCA) and centrifuged at 15,000 g for 15 min. Aliquot (0.5 mL) of the supernatant was mixed with 0.5 mL of 10 mM potassium-phosphate buffer (pH = 7.0) and 1 mL of 1 M potassium iodide. The H_2_O_2_ concentration was evaluated using standards of 5–1,000 μM of H_2_O_2_ and calibration curve plotted accordingly. The absorbance of samples and standards was measured at 390 nm and results were expressed as μmol H_2_O_2_/g Fwt.

Lipid peroxidation was assessed according to Azevedo Neto et al. (2006) and measured in terms of malondialdeyde content (MDA). Leaf tissue (0.2 g) was homogenized in 0.1% TCA and the extract was centrifuged at 15,000 g for 10 min. The reaction mixture of 0.5 mL extract and 1.5 mL of 0.5% thioarbituric acid (TBA) in 20% TCA was incubated at 95°C for 25 min and then cooled on ice bath. The absorbance was determined at 532 nm and corrected for non-specific absorbance at 600 nm. MDA amount was determined using the extinction coefficient of 155 mM/cm. Results were expressed as nmol of MDA/g Fwt.

### Activities of antioxidant enzymes and proline content

Leaf tissue was homogenized in a chilled mortar using ice cold extraction buffer containing 1 mM ethylenediaminetetraacetic acid (EDTA), 1% (w/v) polyvinylpyrrolidone (PVPP), 1 mM phenylmethylsulfonyl fluoride (PMSF), and 0.05% Triton X-100 in 50 mM potassium-phosphate buffer (pH = 7.0) (Chrysargyris et al., 2017b). Protein content in the enzyme extracts was determined according to Bradford assay (Bradford, 1976) using bovine serum albumin (BSA) as a standard.

Catalase (EC 1.11.1.6) and SOD (EC 1.15.1.1) activity were assayed as described previously (Chrysargyris et al., 2017b). Catalase activity was assayed in a reaction mixture (1.5 mL) containing 50 mM K-phosphate buffer (pH = 7.0), 10 mM H_2_O_2_ and an enzyme aliquot. The decomposition of H_2_O_2_ was followed at 240 nm. The results were expressed as CAT units/mg of protein (1 unit = 1 mM of H_2_O_2_ reduction/min). SOD was assayed using the photochemical method. Reaction mixture (1.5 mL) containing 50 mM K-phosphate buffer (pH = 7.5), 13 mM methionine, 75 μM nitro blue tetrazolium (NBT), 0.1 mM EDTA, 2 μM riboflavin and an enzyme aliquot. Reaction started with the addition of riboflavin and placing tubes with the reaction mixture below a light source of two 15-watt fluorescent lamps for 15 min. Reaction stopped by placing the tubes in the dark. Mixtures without enzyme extract developed maximal color (control) and non-irradiated mixture used as blank. The absorbance was determined at 560 nm and activity was expressed as units/mg of protein. One unit of SOD activity was defined as the amount of enzyme required to cause 50% inhibition of the NBT photoreduction rate. Peroxidase activity (EC 1.11.1.6) was determined as described by Tarchoune et al. (2012) following the increase in absorbance at 430 nm. Calculations were performed using the coefficient of extinction of 2.47 mM/cm. One POD unit was defined as the amount of enzyme to decompose 1 μmol of H_2_O_2_ per minute. Results were expressed as units of peroxidase/mg of protein. The activity of APX (EC 1.11.1.11) was determined according to Zhu et al. (2004), by the decrease in absorbance of ascorbate at 290 nm. Results were expressed as units APX/mg of protein.

Proline was measured with the method of acid-ninhydrin and toluene at 520 nm, as described by Khedr et al. (2003). The amount of proline was calculated using a standard curve of proline and results were expressed as μg proline/g Fwt.

### Statistical methods

Data were statistically analyzed using analysis of variance (ANOVA) by IBM SPSS v.22, and presented as treatment mean ± SE of six biological measurements. Pairwise metabolites effects correlations were calculated by Pearson's correlation test using the R program. The components chemical structure and the relationship among treatments were determined by Linear Discriminate analysis (LDA) as described previously (Chrysargyris et al., 2016b), and performed at the percentages of all identified compounds for all treatments by SPSS program. Duncan's multiple range tests were calculated for the significant data at *P* < 0.05.

## Results

### Plant growth

Data presented in Tables 1, 2 indicated that plant growth variables were mainly influenced by salinity and less by mineral foliar applications. Plant height and stem thickness were significantly decreased at NaCl concentrations >50 mM, while no differences were observed at lower salinity levels (i.e., 50 mM NaCl) (Table 1). Also, the salinity conditions (25–50–100 mM NaCl) reduced leaf length, fresh upper biomass and biomass dry matter content up to 16, 53, and 27%, respectively. Middle (50 mM NaCl) and high (100 mM NaCl) salinity reduced root fresh weight while root length was reduced only at a NaCl concentration of 100 mM (Table 2). As a consequence, the ratio of biomass:root was greater at NaCl concentrations >50 mM respect to the low (i.e., 25 mM NaCl) or non-saline treatment, mainly due to the lower root development (lower root fresh weight). No differences were found for shoot number (averaged in 3.54) and root dry matter content (averaged in 11.03%).

**Table 1:** Effect of salinity levels (0–25–50–100 mM NaCl) and foliar applications (no foliar, K, Zn, and Si) on lavender plant height (cm), leaf length (cm), stem thickness (mm), shoot number, biomass fresh weight (FW; g/plant), and biomass dry matter (DM; %) in plants grown hydroponically in perlite.

**Salinity (NaCl)**	**Foliar applic**.	**Plant height**	**Leaf length**	**Stem thickness**	**Shoot number**	**Biomass FW**	**Biomass DM**
0 mM	0	40.83 ± 1.53a^Y^	6.12 ± 0.15ab	5.49 ± 0.15ab	4.33 ± 0.71abc	27.63 ± 1.19a	31.21 ± 0.51a
	+K	36.50 ± 2.04bcd	6.03 ± 0.09abcd	5.40 ± 0.22ab	4.67 ± 0.55ab	20.20 ± 1.52bc	32.94 ± 0.39a
	+Zn	39.00 ± 0.36ab	6.08 ± 0.13abc	5.81 ± 0.31a	5.17 ± 0.65a	20.84 ± 1.66bc	33.21 ± 0.81a
	+Si	38.00 ± 1.29ab	6.15 ± 0.08a	5.47 ± 0.25ab	3.67 ± 0.33abcd	23.58 ± 1.53b	31.98 ± 1.25a
25 mM	0	37.33 ± 1.52abc	5.72 ± 0.16cde	5.37 ± 0.36ab	4.17 ± 0.54abcd	23.54 ± 2.07b	25.38 ± 1.28bc
	+K	33.00 ± 1.41de	5.75 ± 0.18bcde	5.38 ± 0.37ab	3.83 ± 0.54abcd	20.44 ± 1.50bc	27.32 ± 0.41b
	+Zn	33.67 ± 1.28cde	5.68 ± 0.11def	4.82 ± 0.19bcd	3.67 ± 0.71abcd	19.71 ± 1.33bc	27.39 ± 1.36b
	+Si	31.83 ± 1.53ef	5.67 ± 0.18def	5.14 ± 0.20abc	4.67 ± 0.33ab	20.32 ± 1.17bc	27.50 ± 1.24b
50 mM	0	27.67 ± 0.95g	5.32 ± 0.09fgh	4.31 ± 0.21de	3.67 ± 0.42abcd	18.37 ± 0.81cd	24.80 ± 0.71bc
	+K	28.83 ± 1.16fg	5.40 ± 0.12efg	4.55 ± 0.15cde	3.50 ± 0.88abcd	17.34 ± 1.23cd	25.17 ± 0.98bc
	+Zn	27.00 ± 1.39g	5.05 ± 0.11gh	4.32 ± 0.27de	2.67 ± 0.21cdef	15.59 ± 1.31de	25.31 ± 0.22bc
	+Si	26.50 ± 0.72g	5.05 ± 0.05gh	4.57 ± 0.19cde	3.50 ± 0.42abcd	19.69 ± 1.08bc	23.67 ± 0.91cd
100 mM	0	21.17 ± 1.08h	5.13 ± 0.04gh	3.93 ± 0.20e	3.33 ± 0.21bcde	13.03 ± 1.09ef	22.71 ± 0.93cd
	+K	19.58 ± 1.34h	5.02 ± 0.19gh	3.19 ± 0.21f	1.50 ± 0.34f	9.72 ± 0.87f	21.25 ± 0.85d
	+Zn	21.17 ± 2.16h	4.63 ± 0.06i	2.98 ± 0.18f	1.83 ± 0.40ef	11.63 ± 0.87ef	21.02 ± 0.87d
	+Si	19.92 ± 0.71h	4.97 ± 0.06hi	2.85 ± 0.10f	2.50 ± 0.34ef	11.68 ± 0.80ef	22.61 ± 0.80cd
**SIGNIFICANCE**							
Salinity (S)		^***^	^***^	^***^	^***^	^***^	^***^
Foliar (F)		^*^	ns	ns	ns	^***^	ns
S × F		ns	ns	ns	ns	ns	ns

Y *values (n = 6) in columns followed by the same letter are not significantly different, P < 0.05*.

ns, *, and *** *indicate non-significant or significant differences at P < 5, and 0.1%, respectively, following two-way ANOVA*.

**Table 2:** Effect of salinity levels (0–25–50–100 mM NaCl) and foliar applications (no foliar, K, Zn, and Si) on lavender root fresh weight (FW; g/plant), root dry matter (DM; %), biomass:root ratio, and root length (cm) in plants grown hydroponically in perlite.

**Salinity (NaCl)**	**Foliar applic**.	**Root FW**	**Root DM**	**Biomass:root**	**Root length**
0 mM	0	15.85 ± 0.85a ^Y^	12.16 ± 0.31abcd	1.81 ± 0.13cd	26.50 ± 1.68ab
	+K	15.33 ± 1.60a	10.69 ± 0.42abcd	1.54 ± 0.14d	26.00 ± 1.71ab
	+Zn	12.35 ± 0.52ab	12.61 ± 0.93abc	2.01 ± 0.15cd	27.33 ± 1.25a
	+Si	14.93 ± 1.95a	13.29 ± 1.01ab	1.73 ± 0.14cd	27.00 ± 1.63ab
25 mM	0	15.33 ± 1.28a	9.09 ± 1.85d	1.88 ± 0.10cd	27.00 ± 0.96ab
	+K	14.63 ± 2.00a	11.20 ± 0.57abcd	1.60 ± 0.13cd	24.50 ± 1.87abc
	+Zn	12.25 ± 1.61ab	9.94 ± 1.49cd	1.92 ± 0.24cd	27.33 ± 1.85a
	+Si	13.23 ± 0.68a	9.27 ± 0.56d	1.65 ± 0.17cd	23.67 ± 0.61abc
50 mM	0	7.38 ± 1.06cd	13.44 ± 0.82a	2.79 ± 0.32ab	22.67 ± 1.68bcd
	+K	9.17 ± 0.78bc	9.45 ± 0.57d	2.29 ± 0.22abcd	22.83 ± 1.01bcd
	+Zn	6.90 ± 0.45cd	12.12 ± 1.29abcd	2.39 ± 0.34abc	22.83 ± 1.27bcd
	+Si	9.22 ± 0.61bc	9.89 ± 0.48cd	2.05 ± 0.15bcd	21.33 ± 1.05cde
100 mM	0	5.38 ± 0.69d	10.52 ± 0.36abcd	2.96 ± 0.34a	16.75 ± 0.96f
	+K	5.07 ± 0.58d	10.40 ± 0.56abcd	2.37 ± 0.36abc	18.92 ± 0.89def
	+Zn	4.38 ± 0.68d	12.01 ± 0.74abcd	2.78 ± 0.29ab	17.83 ± 0.98ef
	+Si	4.33 ± 0.81d	10.33 ± 0.86bcd	2.88 ± 0.26a	16.83 ± 0.79f
**SIGNIFICANCE**					
Salinity (S)		^***^	^**^	^***^	^***^
Foliar (F)		^*^	ns	ns	ns
S × F		ns	^*^	ns	ns

Y *values (n = 6) in columns followed by the same letter are not significantly different, P ≤ 0.05*.

ns, *, **, and *** *indicate non-significant or significant differences at P < 5, 1, and 0.1%, respectively, following two-way ANOVA*.

Considering the effects of foliar mineral application on the plant growth, the application of K, Zn, and Si reduced the fresh upper biomass in non-saline treatments, whereas this was not evident in saline treatments (Table 1). The application of K at NaCl concentration of 0 and 25 mM (including Si) reduced plant height. Interestingly, foliar application of K, Zn, and Si at highest (100 mM NaCl) salinity reduced further the stem thickness and shoot number produced. The application of K at a NaCl concentration of 50 mM reduced the root dry matter content compared to the relevant control treatment (50 mM NaCl without foliar application) (Table 2).

Summarizing in Tables 1, 2, two-way ANOVA revealed that salinity significantly (*P* < 0.01; *P* < 0.001) affected plant growth parameters, both in upper and root part of the plant, while foliar application affected plant height (*P* < 0.05); biomass fresh weight (*P* < 0.001); and root fresh weight (*P* < 0.05). The interaction of salinity × foliar application affected (*P* < 0.05) root dry matter content through salinity impacts.

### Physiological parameters

Examining the effect of salinity and/or foliar mineral application on physiological parameters, it was found that salinity at NaCl concentrations >50 mM reduced (up to 73%) the content of Chl a and Chl b, and as a consequence, the total Chl content (Table 3). In general, foliar application did not have any profound effect on chlorophyll content, with the exception of the Si treatment at a NaCl concentration of 25 mM, that caused reduction in Chl a, Chl b, and total Chl of 28, 29, and 28%, respectively. However, neither salinity nor minerals affected leaf stomatal conductivity (averaged in 1.51 cm/s). Two-way ANOVA revealed that salinity affected (*P* < 0.001) the content of chlorophylls, while neither the foliar application nor the salinity × foliar interaction affected physiological parameters in any way.

**Table 3:** Effect of salinity levels (0–25–50–100 mM NaCl) and foliar applications (no foliar, K, Zn, and Si) on lavender leaf stomatal conductivity (cm/s), chlorophylls (Chl a, Chl b, Total Chl) content (mg/g fresh weight) in plants grown hydroponically in perlite.

**Salinity (NaCl)**	**Foliar applic**.	**Stomatal conductivity**	**Chl a**	**Chl b**	**Total Chl**
0 mM	0	1.25 ± 0.44a ^Y^	1.60 ± 0.10a	0.55 ±0.04a	2.15 ±0.14a
	+K	1.66 ± 0.42a	1.62 ± 0.07a	0.56 ± 0.02a	2.19 ± 0.09a
	+Zn	1.38 ± 0.56a	1.52 ± 0.07ab	0.53 ± 0.02a	2.06 ±0.10ab
	+Si	1.00 ± 0.45a	1.37 ± 0.13abc	0.48 ± 0.05ab	1.85 ±0.19abc
25 mM	0	1.21 ± 0.62a	1.31 ± 0.12abc	0.45 ± 0.05ab	1.77 ±0.17abc
	+K	1.71 ± 0.29a	1.15 ± 0.12cde	0.36 ± 0.04bcd	1.52 ±0.16cde
	+Zn	2.25 ± 0.40a	1.21 ± 0.09bcd	0.41 ± 0.03bc	1.61 ±0.12bcd
	+Si	2.11 ± 0.43a	0.95 ± 0.14def	0.32 ± 0.05cd	1.27 ±0.19def
50 mM	0	1.31 ± 0.58a	0.81 ± 0.12efg	0.25 ± 0.04def	1.07 ±0.17efgh
	+K	1.63 ± 0.51a	0.94 ± 0.10def	0.30 ± 0.03cde	1.24 ±0.13def
	+Zn	2.12 ± 0.61a	0.75 ± 0.09fgh	0.25 ± 0.03def	1.01 ±0.13fgh
	+Si	0.93 ± 0.45a	0.87 ± 0.17def	0.29 ± 0.05cde	1.16 ±0.23defg
100 mM	0	1.15 ± 0.23a	0.42 ± 0.05h	0.14 ± 0.01f	0.57 ± 0.06i
	+K	1.74 ± 0.70a	0.71 ± 0.10fgh	0.24 ± 0.03def	0.96 ±0.13fghi
	+Zn	0.87 ± 0.30a	0.52 ± 0.20gh	0.18 ± 0.02ef	0.71 ±0.10ghi
	+Si	1.77 ± 0.56a	0.45 ± 0.08h	0.15 ± 0.02f	0.60 ±0.10hi
**SIGNIFICANCE**					
Salinity (S)		ns	^***^	^***^	^***^
Foliar (F)		ns	ns	ns	ns
S × F		ns	ns	ns	ns

Y *values (n = 6) in columns followed by the same letter are not significantly different, P < 0.05*.

ns and *** *indicate non-significant or significant differences at P < 0.1%, respectively, following two-way ANOVA*.

**Figure 1:**
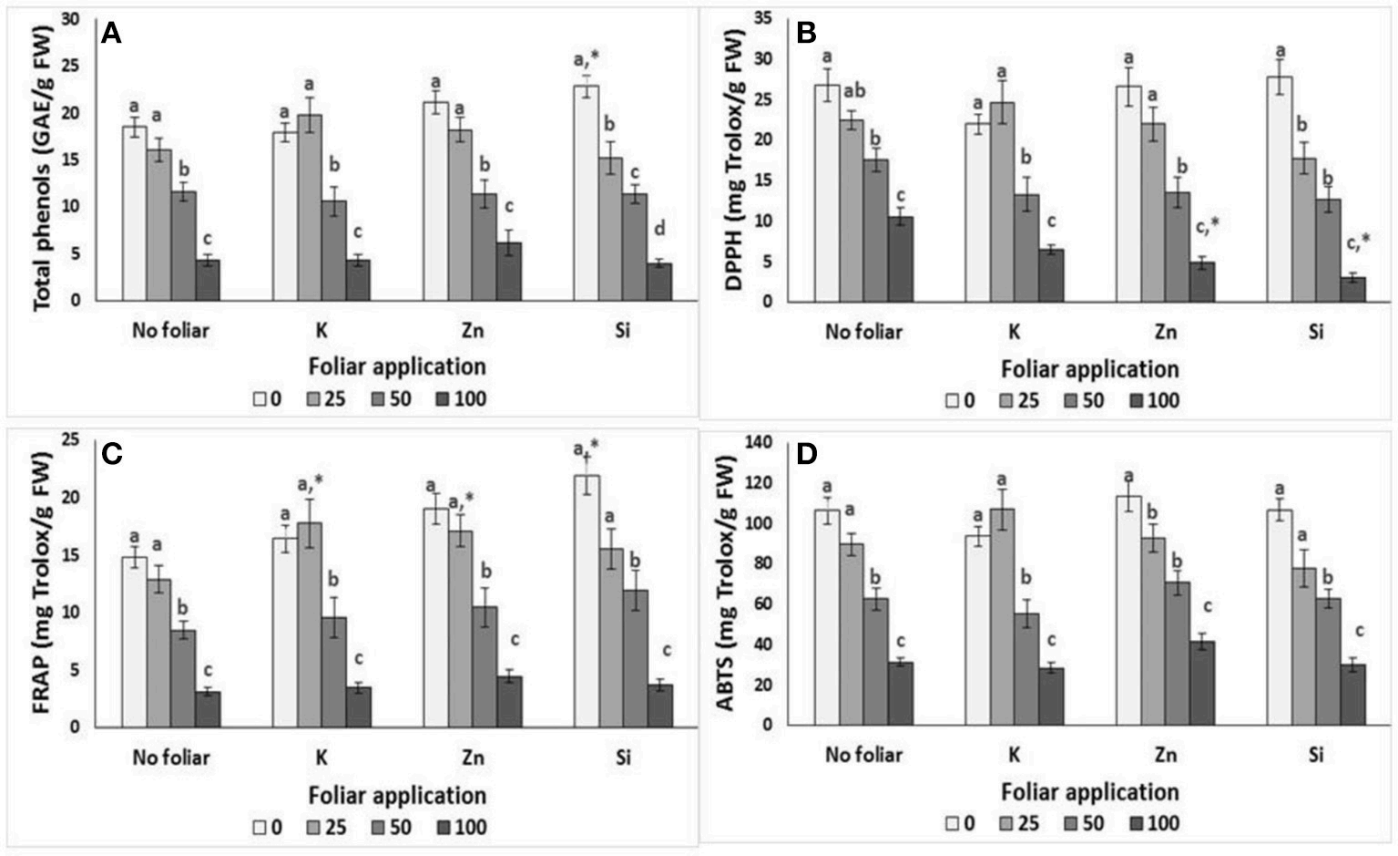
Effect of salinity levels (0–25–50–100 mM NaCl) and foliar applications (no foliar, K, Zn, Si) on the content of total phenols and antioxidant activity in lavender. **(A)** Total phenols, **(B)** DPPH, **(C)** FRAP, and **(D)** ABTS. Significant differences (*P* < 0.05) among salinity treatments are indicated by different letters. Star (*) symbol indicated significance differ among no foliar and equivalent cation foliar. Error bars show SE (*n* = 6).

The application of salinity affected the content of total phenols and antioxidative activity of lavender plants (Figure 1). Thus, total phenols, FRAP and ABTS radical scavenging activity were significantly reduced at NaCl concentrations >50 mM comparing with the control (0 mM NaCl) and 25 mM NaCl applications. The same results were obtained for the DPPH radical scavenging activity respect to the control and NaCl concentrations >50 mM.

K application gave similar results following salinity effects. i.e., the application of NaCl concentrations >50 mM caused total phenols and antioxidant activity reductions. However, foliar with K increased the FRAP activity compared to relevant control at low (25 mM) salinity treatments but not at middle (50 mM) and high (100 mM NaCl) salinity (Figure 1).

The application of Zn on lavender reduced the total phenols, DPPH and FRAP radical scavenging activity at NaCl concentrations >50 mM respect to the 0 and 25 mM NaCl applications, while this reduction was evident even in low (25 mM NaCl) salinity levels for the ABTS activity (Figure 1). Similar to K application, Zn foliar increased the FRAP activity at NaCl concentration of 25 mM. However, it's worth noting that Zn application at NaCl concentration of 100 mM reduced DPPH radical scavenging activity further.

The FRAP and ABTS activity were reduced at NaCl concentrations >50 mM with Si application, while the DPPH activity and total phenols were reduced even at 25 mM NaCl treatment (Figure 1). Si application increased FRAP activity and total phenols in lavender plants grown in non-saline conditions. However, similar to Zn foliar, Si application at NaCl concentration of 100 mM reduced further the DPPH radical scavenging activity.

Two way ANOVA revealed that salinity significantly (*P* > 0.001) affected total phenols and antioxidant activity in lavender, while foliar application affected DPPH (*P* < 0.05) and FRAP (*P* < 0.001) activity (Table 4). Salinity × foliar interaction affected (*P* < 0.05) the content of total phenols and DPPH activity, resulting by the salinity treatment impacts.

**Table 4:** Effect of salinity levels (0–25–50–100 mM NaCl) and foliar applications (no foliar, K, Zn, and Si) on the content of total phenols and antioxidant activity in lavender grown hydroponically in perlite.

**Significance**	**Total Phenols**	**DPPH**	**FRAP**	**ABTS**
Salinity (S)	^***^	^***^	^***^	^***^
Foliar (F)	ns	^*^	^**^	ns
S × F	^*^	^*^	ns	ns

ns, *, **, and *** *indicate non-significant or significant differences at P ≤ 5, 1, and 0.1%, respectively, following two-way ANOVA*.

**Figure 2:**
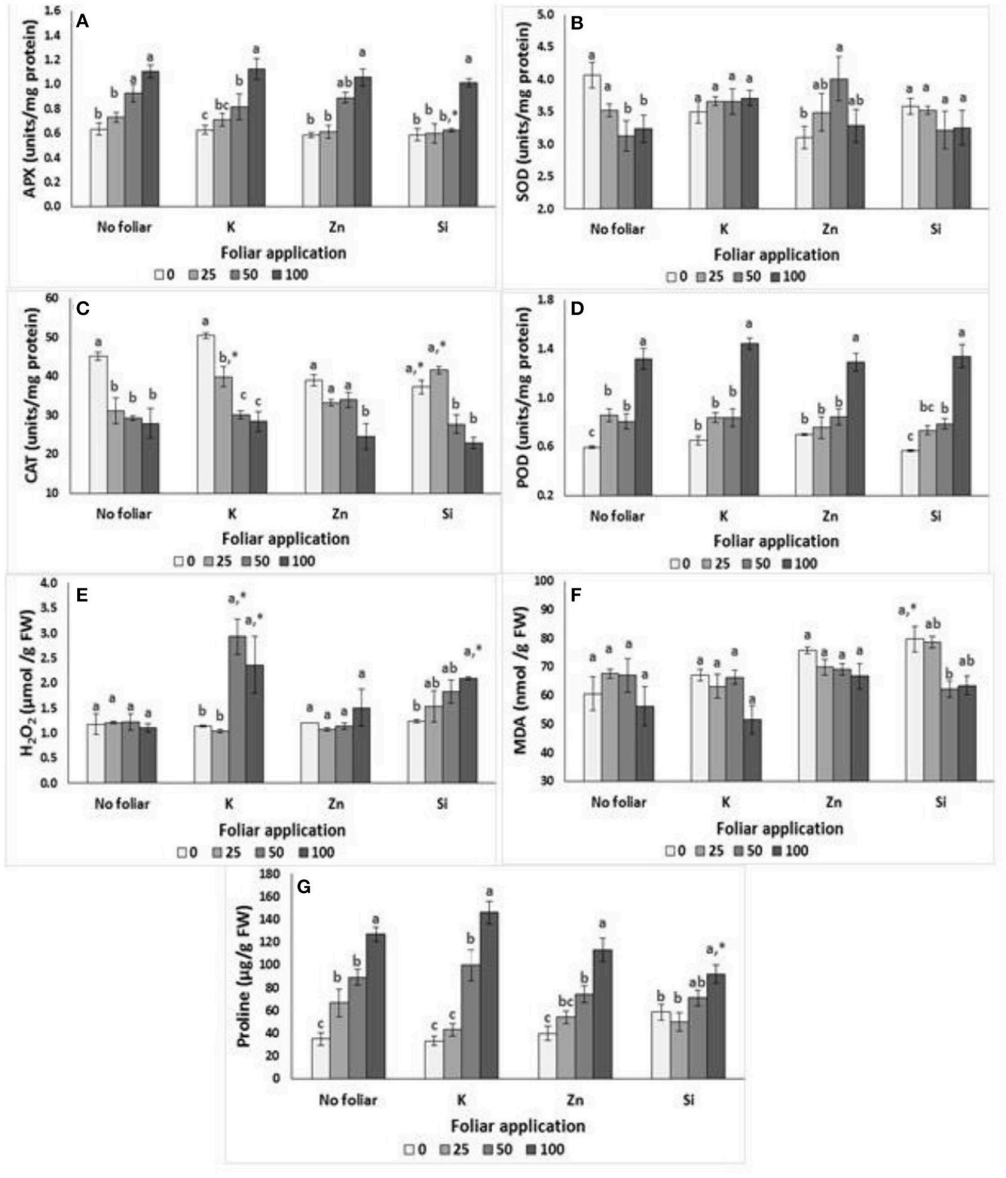
Effect of salinity levels (0–25–50–100 mM NaCl) and foliar applications (no foliar, K, Zn, Si) on the damage index and antioxidant enzymes activities in lavender. **(A)** APX, **(B)** SOD, **(C)** CAT, **(D)** POD, **(E)** H_2_O_2_, **(F)** Lipid peroxidation (MDA), and **(G)** proline. Significant differences (*P* < 0.05) among salinity treatments are indicated by different letters. Star (^*^) symbol indicated significance differ among no foliar and equivalent cation foliar. Error bars show SE (*n* = 6).

The effects of salinity into nutrient solution on damage index, enzymes activity and proline content in lavender plants are presented in Figure 2. The APX activity increased at NaCl concentrations >50 mM, while the opposite occurred in SOD activity. Moreover, the increased salinity levels affected the proline content and POD activity, with greater values to be found at NaCl concentration of 100 mM, while no differences were found among 25 and 50 mM of NaCl treatment. Plants grown under salinity had lower CAT values, which were independent of the salinity levels applied. Neither H_2_O_2_ nor MDA production got significantly affected by salinity treatments (Figures 2E,F).

APX activity and proline content were increased but CAT activity was reduced in plants grown under salinity and K foliar (Figures 2A,C,G). Indeed, the K application increased the CAT activity in 25 mM NaCl-treated plants comparing to the no foliar treatment (Figure 2C). POD activity did not differ in plants grown in salinity < 50 mM NaCl+K while POD activity almost doubled in 100 mM of NaCl+K. The H_2_O_2_ production significantly increased in plants grown at NaCl concentrations >50 mM. K application increased the H_2_O_2_ production in 50 and 100 mM NaCl-treated plants comparing to no foliar treatments. No significant differences were found on SOD activity and MDA production.

Considering the Zn foliar application, the APX activity was increased (up to 80%) in plants grown at NaCl concentration 100 mM comparing with the activity of plants grown at NaCl concentration 25 mM or non-saline treatment (Figure 2). Additionally, plant grown at NaCl concentrations <50 mM did not differ on POD values (despite the increased tendency related to the increased salinity levels) and CAT values (despite the reduced tendency related to the increased salinity levels). However, the Zn application at a NaCl concentration of 100 mM, resulted in significant higher POD value and lower CAT value comparing with the control treatments (no foliar). Similar to K, the Zn application increased the proline content in relation to the salinity level increments. Neither H_2_O_2_ nor MDA production got significantly affected by salinity treatments (Figures 2E,F). SOD activity varied among Zn treatments without specific trend for the Zn effect.

Foliar application of Si on plants grown under different salinity levels had similar effects with the application of K as mentioned previously (Figure 2). Therefore, APX remained in similar levels in Si-treated plants grown at NaCl concentrations <50 mM, but increased at NaCl concentration of 100 mM. Interestingly, Si foliar reduced the APX values at NaCl concentration of 50 mM comparing with the no foliar treatment. CAT activity was reduced in plants grown under moderate salinity (>50 mM NaCl). Indeed, the Si application increased the CAT activity in 25 mM NaCl-treated plants comparing to the no foliar treatment. To the contrary, Si application reduced CAT activity in non-saline treated plants comparing to the no foliar treatment. POD activity increased in plants grown in different salinity levels with greater (two-times) values to be found at NaCl concentration 100 mM. The H_2_O_2_ production significantly increased in Si-treated plants grown at NaCl concentration 100 mM respect to the non-saline treatment. No significant differences were found on SOD activity among treatments. MDA production varied among Si treatments without specific trend for the Si effect. However, the Si foliar application increased the MDA production in plants grown in non-saline conditions comparing to the no foliar treatment. The proline content following Si foliar was increased at NaCl concentration of 100 mM comparing with 0–25 mM NaCl+Si.

Two-way ANOVA revealed that both salinity (*P* < 0.001) and foliar (*P* < 0.01) but not their interaction, affected APX activity. CAT activity and H_2_O_2_ production were significantly affected either by salinity, foliar or their interaction (salinity × foliar) as presented in Table 5. MDA activity was affected by salinity (*P* < 0.05) or foliar (*P* < 0.05) application, but not by their interaction. Salinity affected (*P* < 0.001) POD activity, while foliar application did not cause any significant effect. Salinity affected (*P* < 0.001) the content of proline, and this effect was also evident in saline × foliar interaction. Interestingly, neither salinity nor foliar application affected SOD content, while their combined effect (salinity × foliar interaction) resulted in significant (*P* < 0.05) impact.

**Table 5:** Effect of salinity levels (0–25–50–100 mM NaCl) and foliar applications (no foliar, K, Zn, and Si) on the damage index and antioxidant enzymes activities in lavender grown hydroponically in perlite.

**Significance**	**APX**	**SOD**	**CAT**	**POD**	**MDA**	**H_2_O_2_**	**Proline**
Salinity (S)	^***^	ns	^***^	^***^	*	^**^	^***^
Foliar (F)	^**^	ns	^*^	ns	^*^	^***^	ns
S × F	ns	^*^	^**^	ns	ns	^**^	^**^

ns, *, **, and *** *indicate non-significant or significant differences at P ≤ 5, 1, and 0.1%, respectively, following two-way ANOVA*.

### Mineral nutrient content

**Figure 3:**
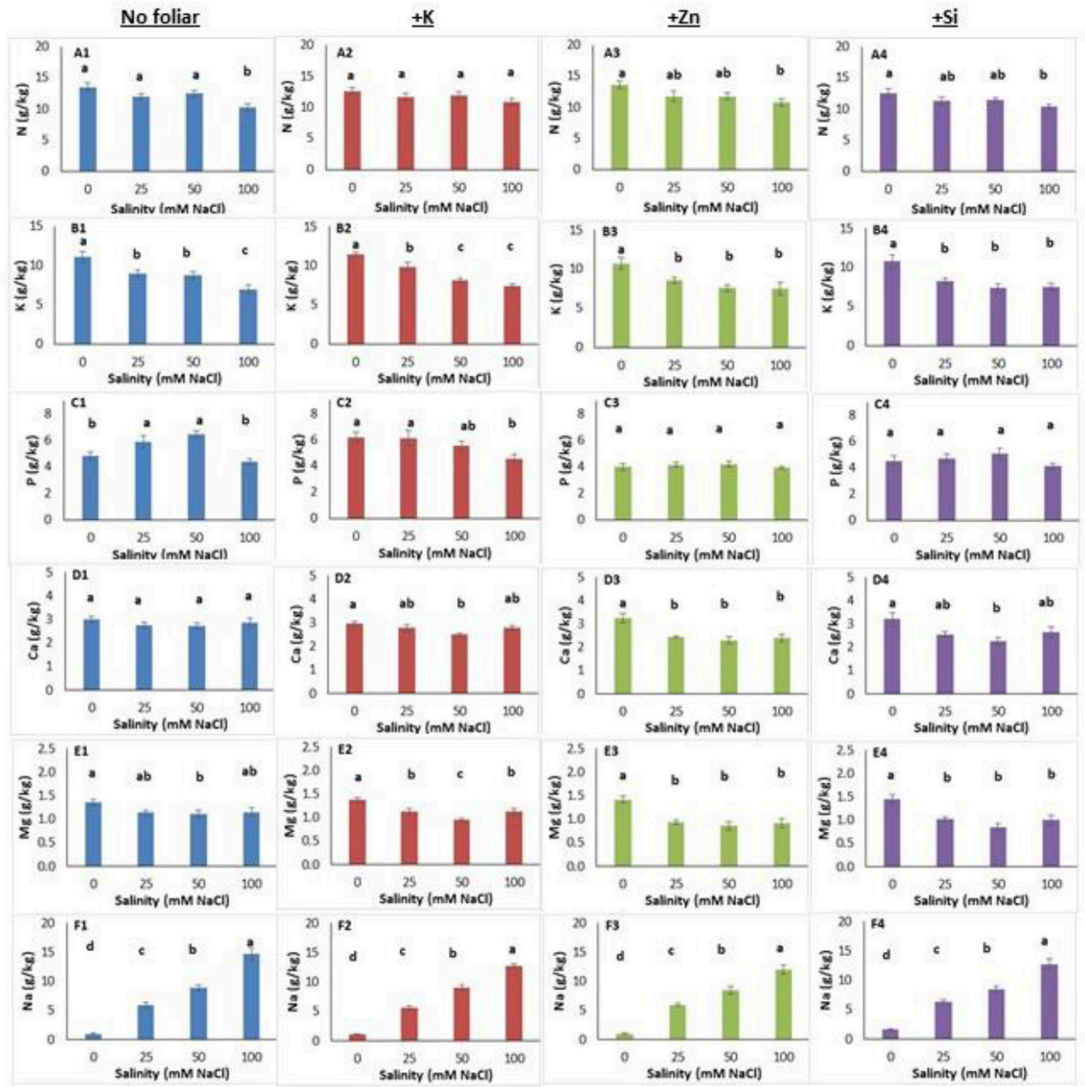
Lavender leaf analysis (macronutrient) of plants grown hydroponically in perlite under different salinity levels (0–25–50–100 mM NaCl) and foliar applications (no foliar, K, Zn and Si). Sub-figures **(A–F)** referring to different macronutrients and numbering **(1–4)** referring to the no foliar, K, Zn and Si, respectively. Significant differences (*P* < 0.05) among treatments are indicated by different letters. Error bars show SE (*n* = 6).

**Figure 4:**
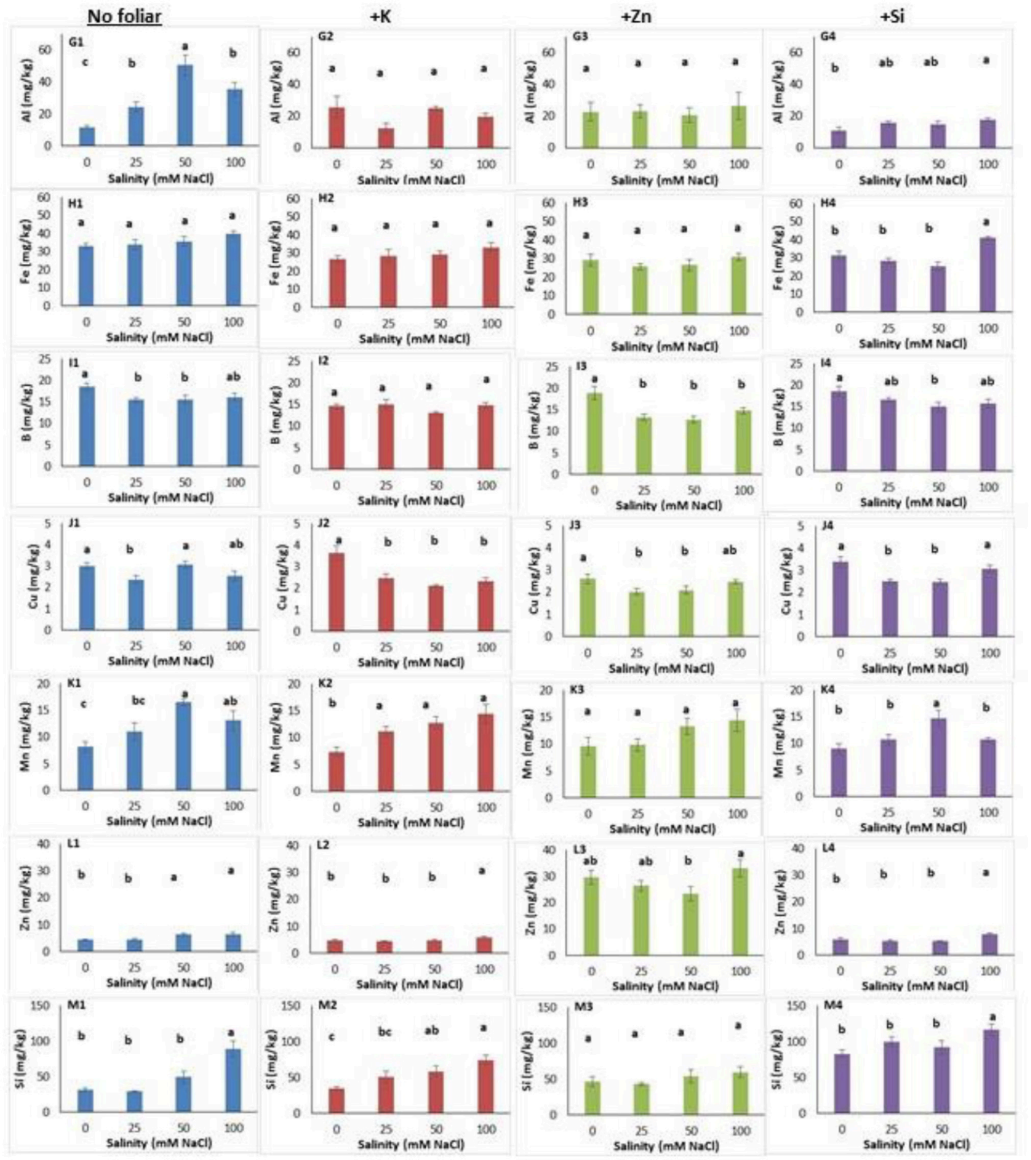
Lavender leaf analysis (micronutrient) of plants grown hydroponically in perlite under different salinity levels (0–25–50–100 mM NaCl) and foliar applications (no foliar, K, Zn and Si). Sub-figures **(G–M)** referring to different micronutrients and numbering **(1–4)** referring to the no foliar, K, Zn and Si, respectively. Significant differences (*P* < 0.05) among treatments are indicated by different letters. Error bars show SE (*n* = 6).

Salinity level and foliar application affected micro- and macro-nutrient content in both leaves and roots (Figures 3, 4, Figure S2). In leaves, salinity at a NaCl concentration of 100 mM reduced (up to 24%) nitrogen content (Figure 3A1). Foliar K application alleviated the high salinity negative effect as the leaf nitrogen content was similar among treatments (Figure 3A2). The Zn and Si foliar application reduced nitrogen content at NaCl concentration of 100 mM compared with the non-saline treatments (Figures 3A3,A4). The leaf potassium content decreased (up to 37%) in plants grown under salinity (Figure 3B1) while neither K (Figure 3B2) nor Zn and Si application alleviated the adverse salinity effects (Figures 3B3,B4). Leaf phosphorus content was increased (5.89 and 6.47 g/kg tissue) in plants grown within NaCl concentrations of 25–50 mM, respectively (Figure 3C1). The K application reduced the leaf phosphorus content at NaCl concentration of 100 mM (Figure 3C2). The foliar application with Zn or Si did not change the leaf phosphorus content (Figures 3C3,C4). Salinity did not affect (Figure 3D1) but Zn application reduced leaf calcium content in saline-grown plants (Figure 3D3). However, K or Si application reduced leaf calcium content at NaCl concentration of 50 mM (Figures 3D2,D4). Magnesium content in leaves was reduced in plants grown at NaCl concentration of 50 mM (Figure 3E1). Cation application reduced the leaf magnesium content in saline-treated plants (Figures 3E2–E4). As expected, increasing salinity levels resulted in increasing sodium content in leaves (Figure 3F1). The same trend was found in plants grown under saline with the foliar (K, Zn, Si) application (Figures 3F2–3F4).

Salinity increased aluminum content in leaves and reached the highest content at a NaCl concentration of 50 mM (Figure 4G1). Indeed, K and Zn foliar application alleviated the aluminum inductions in saline-treated plants, as the aluminum content did not differ among plants grown in saline and non-saline conditions (Figures 4G2,G3). However, the Si application posed same effects, to relieve aluminum content in leaves only at NaCl concentrations of 25 and 50 mM, but not at 100 mM (Figure 4G4). Neither salinity nor K or Zn application affected iron content in leaves (Figures 4H1–4H3) while Si application increased iron content in plants grown at NaCl concentration 100 mM (Figure 4H4). The leaf boron content reduced in plants grown at NaCl concentrations 25 and 50 mM compared to the control plants (Figure 4I1). Zn application reduced boron content in plants grown under salinity, and this was evidenced only for the NaCl concentration 50 mM with Si application (Figures 4I3,I4). The application of Zn and Si reduced the copper content in leaves at NaCl concentrations 25 and 50 mM (Figures 4J3,J4) while this was also found for the highest salinity level (100 mM NaCl) following K application (Figure 4J2). No clear observation can be stated for the effect of salinity on leaf copper content (Figure 4J1). Plants grown at NaCl concentration of 50 mM (including Si application) increased manganese content (Figures 4K1,K4). K or Zn application in saline-treated plants did not affect manganese content in leaves (Figures 4K2,K3). The zinc content in leaves increased in plants grown at NaCl concentrations >50 mM (Figure 4L1). Both the application of K or Si increased the zinc content at NaCl concentration of 100 mM (Figures 4L2,L4). Zn foliar application, as expected, increased (up to 5.8 times) the zinc leaf content comparing to no foliar applications (Figure 4L3). Plants grown at a NaCl concentration of 100 mM had higher silicon content (Figure 4M1). Additionally, K application increased silicon content in plants grown at NaCl concentration of 100 mM comparing with plants grown at 25 mM and non-saline (control) conditions (Figure 4M2). Zn application did not alter the silicon content in leaves (Figure 4M3). The Si application increased the silicon content in plants grown at NaCl concentration of 100 mM (Figure 4M4), similar trend as it was found for non-foliar applications (see Figure 4M1). Obviously, Si application increased (up to 1.5 times) the silicon content in leaves, ranged from 83.29 to 116.70 mg/kg.

Following two-way ANOVA, salinity significantly affected (*P* < 0.01; *P* < 0.001) the leaf macro- and micronutrients (except for aluminum), while foliar application significantly affected the content of phosphorus, aluminum, iron, copper, zinc and silicon at level of *P* < 0.001, the content of boron at level of *P* < 0.01 and magnesium content at level of *P* < 0.05 (Table 6). Salinity × foliar interaction affected mainly micronutrients content, such as aluminum, copper and silicon at level of *P* < 0.05, 0.01, and 0.05 respectively.

**Table 6:** Effect of salinity levels (0–25–50–100 mM NaCl) and foliar applications (no foliar, K, Zn, and Si) on the leaf analysis in lavender grown hydroponically in perlite.

**Significance**	**N**	**K**	**P**	**Ca**	**Mg**	**Na**	
**Macronutrients**							
Salinity (S)	^***^	^***^	^***^	^***^	^***^	^***^	
Foliar (F)	ns	ns	^***^	ns	^*^	ns	
S × F	ns	ns	ns	ns	ns	ns	
**Significance**	**Al**	**Fe**	**B**	**Cu**	**Mn**	**Zn**	**Si**
**Micronutrients**							
Salinity (S)	ns	^***^	^***^	^***^	^***^	^**^	^***^
Foliar (F)	^***^	^***^	^**^	^***^	ns	^***^	^***^
S × F	^*^	ns	ns	^**^	ns	ns	^*^

ns, *, **, and *** *indicate non-significant or significant differences at P ≤ 5, 1, and 0.1%, respectively, following two-way ANOVA*.

The effects of salinity and foliar application on root mineral content is presented in Figure S2. Salinity (NaCl) decreased potassium and calcium (at 100 mM), iron and silicon (at ≥ 25 mM), boron (at ≥ 50 mM) but increased phosphorous, sodium, manganese and zinc content in roots. Plants grown under salinity with K application decreased root content for potassium and silicon (at ≥ 25 mM), calcium, boron, copper and iron (at ≥ 50 mM), aluminum (at 100 mM) but increase phosphorus (at 100 mM), sodium and manganese (at ≥ 50 mM) root content compared to control treatment. In case of Zn application, plants grown under salinity decreased root content for potassium, calcium and boron (at ≥ 50 mM), iron (at 100 mM), silicon (at ≥ 25 mM) but increase phosphorus and zinc (at ≥ 50 mM), as well as sodium (at ≥ 25 mM) root content respect to the equivalent control treatment. Plants grown under salinity with Si application decreased root content for potassium, magnesium, silicon and boron (at ≥ 25 mM), but increase nitrogen (at 50 mM), phosphorus, manganese and zinc (at ≥ 50 mM), and sodium (at 100 mM) content in roots.

Following two-way ANOVA, salinity affected (*P* < 0.01; *P* < 0.001) the root macro- and micronutrients (except for aluminum) significantly, while foliar application significantly affected the content of potassium at level *P* < 0.01 and the content of zinc at level of *P* < 0.05 (Table S1). Indeed, the interaction of salinity × foliar application did not cause any effect on root mineral content.

### Essential oil yield and constituents

**Figure 5:**
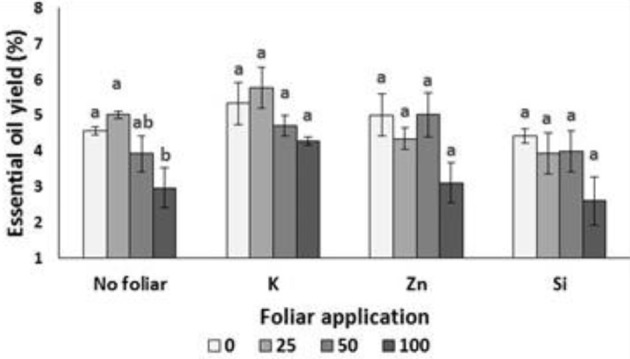
Effect of salinity levels (0–25–50–100 mM NaCl) and foliar applications (no foliar, K, Zn, and Si) on essential oil yield (%) in lavender plant grown hydroponically in perlite. Significant differences (*P* < 0.05) among treatments are indicated by different letters. Error bars show SE (*n* = 6).

Lavender EO yield was reduced in plants grown under NaCl concentration of 100 mM comparing with plants grown in non-saline or low saline levels (Figure 5). The foliar application of minerals (K, Zn, and Si) did not affect the EO yield among treatments, but alleviated the reduced yield which was observed under the high salinity (without any foliar application). Two-way ANOVA revealed that salinity (*P* < 0.01) and foliar application (*P* < 0.05) (but not their interaction) affected the EO yield (Table 7).

**Table 7:** Effect of salinity levels (0–25–50–100 mM NaCl) and foliar applications (no foliar, K, Zn, and Si) on essential oil yield (%) in lavender grown hydroponically in perlite.

**Significance**	**Essential oil yield**
Salinity (S)	^**^
Foliar (F)	^*^
S × F	ns

ns, *, and ** *indicate non-significant or significant differences at P < 5 and 1%, respectively, following two-way ANOVA*.

**Figure 6:**
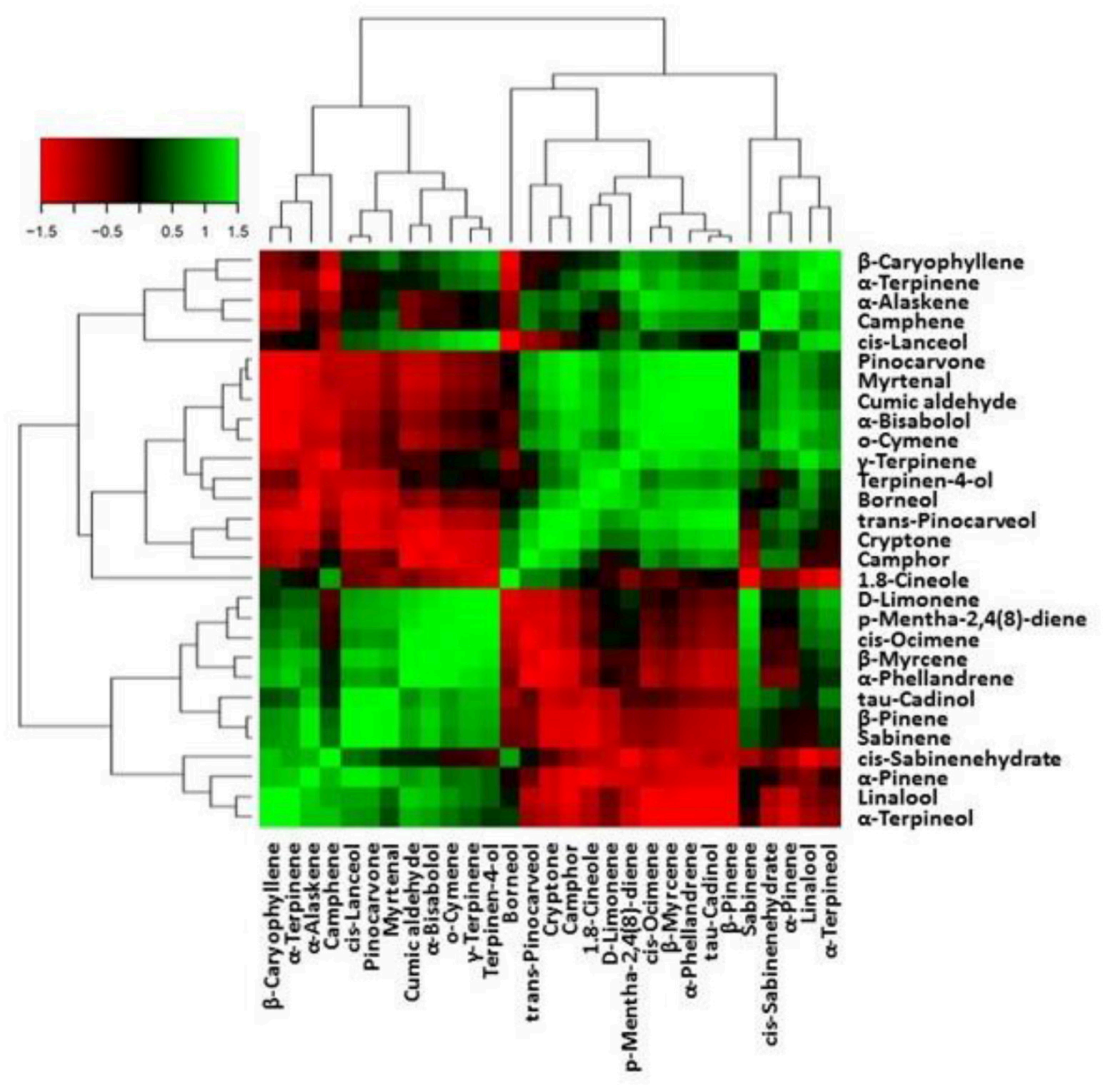
Heat-map matrices of the correlation between EO metabolites in leaves of lavender. Each square indicates *r* (Pearson's correlation coefficient of a pair of metabolites).

The effect of different salinity levels as well as mineral foliar application on chemical composition of the EO of *L. angustifolia* are given in Figure 6 and Tables S2−S5. Considering the EOs analysis, 29 components for salinity, 30 components (excluding o-cymene and including caryophyllene oxide and muurola-5-en-4-one) for K foliar, and 31 (including caryophyllene oxide and muurola-5-en-4-one) components for Zn and Si applications were identified in the EOs of lavender that underwent different treatments that represented 98.86–99.62% of the oils. It can be noticed that, hydrocarbon (monoterpenes and sesquiterpenes) compounds were ranged from 16.74 to 24.80% and 0.48 to 1.13%, respectively, while oxygenated (monoterpenes and sesquiterpenes) compounds were ranged from 69.58 to 79.82% and 2.13 to 4.19%, respectively (Tables S2−S5). The major components were 1.8-cineole (alcohol), D-limonene (monoterpene hydrocarbon), β-pinene (monoterpene hydrocarbon), camphor (ketone), α-pinene (monoterpene hydrocarbon), borneol (alcohol), α-terpineol (alcohol), β-myrcene (monoterpene hydrocarbon), sabinene (monoterpene hydrocarbon), *cis*-lanceol (alcohol), and α-bisabolol (alcohol). Other components were present in amounts < 1% in most treatments (Tables S2−S5).

The correlation matrix of EO metabolites is presented in Figure 6. Among metabolites that activated specific terpenoids backbone biosynthesis related to a positive correlation are α-pinene and β-pinene, sabinene through sabinene hydrate, α-terpineol, β-myrcene through linalool, and D-limonene. The above metabolites were negative correlated with 1.8-cineole, or the formation of borneol and camphor thorough (+)-bornyl-diphosphate.

**Figure 7:**
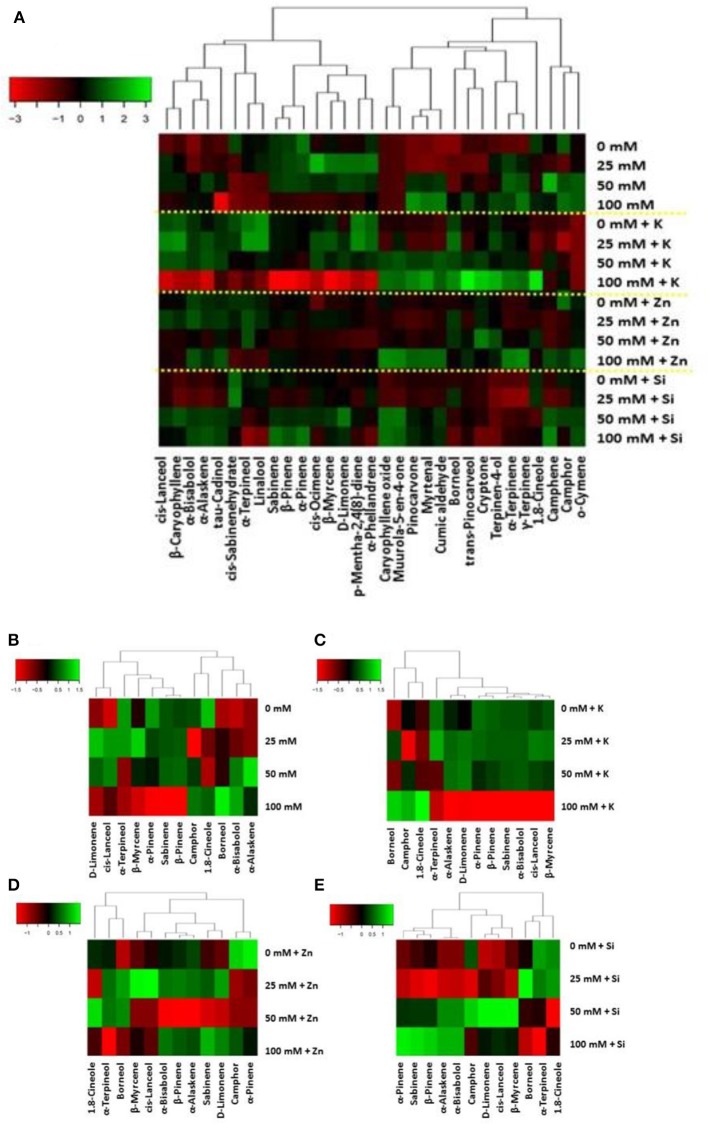
Metabolite changes in leaves of lavender. Heat map representing relative expression of volatiles in total **(A)** elicited in leaf tissue following **(B)** salinity (25–50–100 mM NaCl) and foliar **(C-E)** applications (no foliar, K, Zn, and Si) as compared to control (no saline) plants.

Salinity affected oil constituents as β-myrcene but also α-pinene and α-terpineol (including control treatment) increased in 25 mM NaCl-treated plants compared to 100 mM NaCl application (Table S2; Figure 7). Plants grown at NaCl concentration of 100 mM had significantly higher borneol content. Camphor content was significantly low at NaCl concentration 25 mM comparing with the control or NaCl concentrations 50 mM and 100 mM. Thus, the application of 25 mM NaCl concentration increased monoterpenes hydrocarbons (averaged in 24.80%) compared with the application of NaCl concentration 100 mM (averaged in 21.59%). The foliar K application increased α-pinene (at < 25 mM NaCl) and D-limonene, β-pinene, β-myrcene, sabinene, α-bisabolol and *cis*-lanceol content in plants grown at NaCl concentrations <50 mM (Table S3; Figure 7). Contrarily, 1.8-cineole reached its maximal percentage (62.53%) as a result of NaCl concentration 100 mM. The content of α-terpineol was increased in plants subjected to NaCl concentration 25 mM compared with the plants subjected to higher salinity levels. The foliar application of Zn and Si did cause minor changes in lavender EO composition (Tables S4, S5; Figure 7).

**Figure 8:**
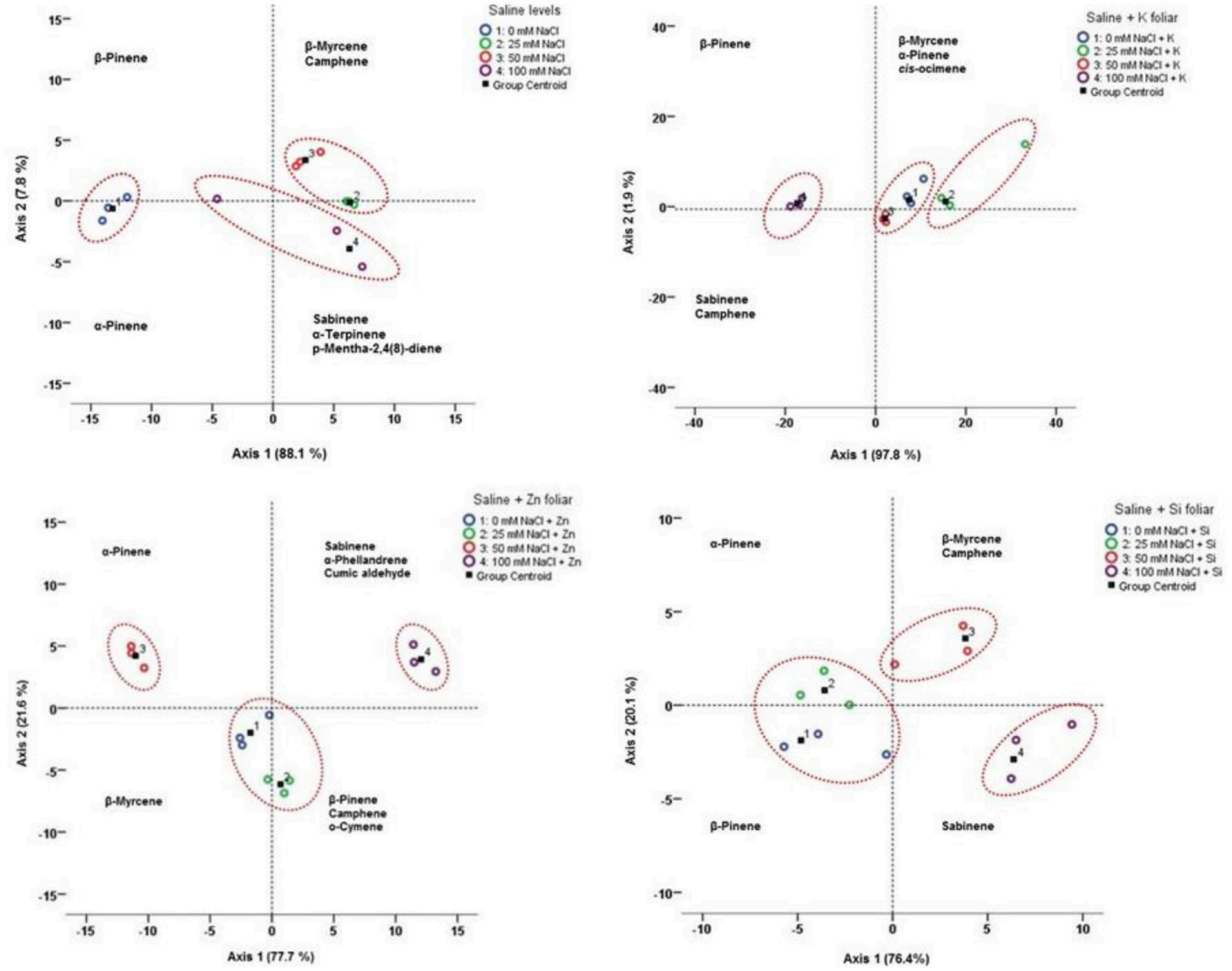
Linear discriminant analysis (LDA) for the lavender essential oil compounds under different saline levels with K, Zn, Si foliar applications. Projection of the average contents of the essential oil compounds onto the first two principal axes (+ and – indicate positive and negative correlations with axes, respectively). Coding numbers referred to mM NaCl + foliar applications.

To identify possible relationships between volatile compounds and salinity with or without the mineral foliar application, LDA was applied for salinity and each individual mineral application (Figure 8). LDA, performed on average contents of all compounds for each salinity level and/or foliar application, showed that the first two principal axes represented 95.9–99.7% of the total variation.

For salinity, the first axis (88.1% of the total variation) was mainly correlated with β-pinene, β-myrcene, camphene, and α-pinene. The second axis represented 7.8% of the total variation, and sabinene, α-terpinene and p-mentha-2,4(8)-diene were the main compounds contributing to its definition. The plot of the projection of the average values of all the compounds onto the first two principal axes, revealed a high chemical dispersion among saline concentrations (0–25–50–100 mM NaCl). Therefore, according to the LDA, three [(a) 0 mM; (b)25 mM; and 50 mM; (c) 100 mM NaCl] concentration groups in relation to the saline levels could be observed.

LDA for salinity and K application showed the first axis (97.8% of the total variation) was mainly correlated with β-myrcene, β-pinene, α-pinene, camphene and *cis*-ocimene. The second axis represented 1.9% of the total variation with sabinene to consist the main compounds contributing to its definition. LDA showed three [(a) 0 mM + K and 50 mM NaCl + K; (b) 25 mM NaCl + K; (c) 100 mM NaCl + K] concentration groups in relation to the saline levels with K foliar could be distinguished.

In case of Zn application LDA showed that the first axis (77.7% of the total variation) was mainly correlated with α-pinene, β-pinene, sabinene, β-myrcene, and o-cymene. The second axis represented 21.6% of the total variation, and camphene, cumic aldehyde and α-phellandrene were the main compounds contributing to its definition. According to the LDA, three [(a) 0 mM + Zn and 25 mM NaCl + Zn; (b) 50 mM NaCl + Zn; (c) 100 mM NaCl +Zn] concentration groups in relation to the saline levels with Zn foliar could be found.

LDA for salinity and Si application showed the first axis (76.4% of the total variation) was mainly correlated with sabinene and β-pinene. The second axis represented 20.1% of the total variation, and β-myrcene, α-pinene and camphene were the main compounds. According to the LDA, three [(a) 0 mM + Si and 25 mM NaCl + Si; (b) 50 mM NaCl + Si; (c) 100 mM NaCl + Si] concentration groups in relation to the saline levels with Zn foliar could be distinguished.

## Discussion

Plants subjected to saline conditions undergo many metabolic changes in order to reduce their ability to absorb water and demonstrate rapid reductions in growth rate. The reduction in growth was attributed to lower osmotic potential in the soil, which leads to decreased water uptake, reduced transpiration and stomata closure (Ben-Asher et al., 2006). The mechanisms of salinity on plant growth are highly related to the following points: (a) salinity affects root and stomatal resistance to water flow, (b) the balance between root and shoot hormones shifts greatly under saline conditions, (c) salinity changes the structure of the chloroplasts and mitochondria and such changes may interfere with normal metabolism and growth, (d) salinity increases respiration and decreases photosynthetic products (Said-Al Ahl and Mahmoud, 2010).

### Plant growth

Plant growth inhibition is a common response to salinity and might be related to salt osmotic effects, which cause cell turgor and expansion (Hendawy and Khalid, 2005). In general, salinity and, to a lesser extent, mineral foliar applications have a pronounced effect on plant growth related parameters. In the present study, levels of salinity at NaCl concentrations >50 mM reduced plant height, stem thickness and root biomass, while on top of that, 25 mM NaCl reduced leaf length, fresh upper biomass and biomass dry matter, being in agreement with previous reports on basil (Tarchoune et al., 2010), lavender and thyme (Cordovilla et al., 2014), chamomile (Nasrin et al., 2012), and parsley (Chondraki et al., 2012). Hejazi Mehrizi et al. (2012) reported that the application of 50 mM of NaCl concentration in the nutrient solution caused only 7% reduction in the shoot and root dry matter production in rosemary, while an equivalent NaCl concentration of 50 mM caused 21% reduction in lavender biomass dry matter indicating that *L. aguistifolia* is less salt tolerant than rosemary. However, Garcia-Caparros et al. (2017) reported that *Lavandula multifida* is better adapted to salinity compared to other members of the Lamiaceae.

Several studies have highlighted the beneficial effects of mineral application either in common plant growing conditions or in saline stressed plants. Considering the effects of foliar mineral application on lavender growth it was found that the application of K, Zn, and Si reduced the fresh upper biomass in non-saline treatments, possibly considered as overdose application of minerals. However, mineral applications alleviated the reduced fresh biomass caused by salinity, as no differences were evident among saline (<50 mM NaCl) and non-saline grown plants with the foliar application. Occasionally, foliar application caused further stress than the individual saline dose, as in the case of K-Zn-Si + 100 mM NaCl application that caused a further decrease in stem thickness and shoot number produced, being unable to alleviate induced stress caused by salinity. Despite the potassium benefits in plant metabolism -promoting carbohydrates synthesis- in the present study, K application did not improve lavender growth, or even reduced plant biomass in non-saline conditions. This might be related to the well balanced nutrient solution as optimized by Chrysargyris et al. (2016b, 2017a), applied in lavender plants under hydroponic conditions. In this sense, K application could be of redundant in the present study. Basil plants sprayed with zinc under normal and saline soils conditions were superior when compared to unsprayed plants (Said-Al Ahl and Mahmoud, 2010). The stimulatory effect of zinc on plant growth was recorded by several researchers (Said-Al Ahl and Omer, 2009) and is attributed to the well-known functions of zinc in CO_2_ assimilation. More specifically, being a component of carbonic anhydrase, several dehydrogenases and auxin production it consequently enhanced the elongation processes (Marschner, 2012). As suggested by Parker et al. (1992) Zn deficiency increased the membrane permeability in root cells indicating the Zn effects in cell membranes. Zn is necessary for root cell membrane integrity and it is this aspect which prevents excessive phosphorus uptake by roots and the translocation of phosphorus from roots to leaves. From this point of view, external Zn concentrations could mitigate the negative effect of NaCl by inhibiting sodium and/or chloride uptake or translocation (Alpaslan et al., 1999). Ramezani et al. (2012) have indicated a trend to alleviate salinity effects by means of iron or iron and zinc application, but no effects on plant growth were noticed after zinc application alone, being in accordance with the present findings.

Silicon has been reported to enhance plant growth particularly under biotic and abiotic stress due to the activation of certain mechanisms. Thus, silicon can increase plant resistance toward salinity stress, which is a major yield limiting factor in arid and semiarid areas. Silicon deposition on the roots exodermis and endodermis reduces Na uptake in plants (Gong et al., 2003). Al-Aghabary et al. (2004) reported dry matter increase in saline environments and indicated beneficial effects of Si application in alleviating salinity stress. The foliar application of 250 up to 1,000 ppm SiO_2_ increased the growth parameters of faba bean in saline soils (Hellal et al., 2012). Foliar application of Si was reported in gerbera (*Gerbera* hybrid L.) with a considerable increase in growth and flower quality related parameters (Kamenidou et al., 2010).

Salinity adaptation can be obtained through Na and Cl elimination which requires either high tissue tolerance to Na and Cl or preventing ions accumulation in plant tissue. Generally, according to Gupta and Gupta (1997), positive interaction between salinity and nutrients is reduced by increasing salinity and what is more, the plant's response to additional fertilization may be neutral or even lead to a reduction of yield, growth etc.

### Physiological parameters

The reduction in growth and chlorophyll level in foliage of lavender plants exposed to salt as compared to control plants follows the pattern observed in several other species (Koocheki et al., 2008; El-Danasoury et al., 2010). The decrease in chlorophylls under salt stress may be due to reduction in pigment biosynthesis or enzymatic chlorophyll degradation (Yang et al., 2009). Lee et al. (2010) reported that Si alleviated the negative effects of NaCl on chlorophyll reduction. However, this alleviating property of Si was not found in the current study, possibly due to the different species (soybean crop), Si concentration (2.5 mM Si) used and Si application through the nutrient solution rather than in the foliar application. The similar leaf stomatal conductance among treatments might be related to the salinity stress harmonization (prevention) by the well-balanced nutrient solution in the current hydroponic study as well as the low mineral needs that lavender crop has in general.

Salinity reduced the content of total phenols as well as the antioxidative activity of the lavender plants. According to Wong et al. (2006) photosynthesis is declined (chlorophyll content as well) due to disturbance of enzymatic activities after salinity application, and as a result, polyphenols decrease. As a result, antioxidant activity is reduced too. Basil grown in saline (50 mM NaCl) conditions did not demonstrate any changes in the antioxidant activity of plant tissue (Tarchoune et al., 2010), being in agreement with the present study, in terms of lower salt concentration i.e., 25 mM NaCl. In coriander fruits, polyphenol content decreased in response to salinity (Neffati et al., 2011).

Phenolics are well-known antioxidants acting as powerful radical scavengers and ions chelators (Balasundram et al., 2006). The phenolics content increased under salinity in response to the oxidative stress generated (prevention of stress-induced oxidative damage or maintenance of osmotic balance) by the formation of ROS in these hostile environments (Navarro et al., 2006). In the DPPH system, the antioxidant activity in control plants was superior to all the examined samples. Under saline conditions, the scavenging activity was strongly diminished in comparison to the control. Our results are similar to those obtained in coriander under saline conditions, as in that study it was suggested that in coriander fruits an imbalance between ROS generation and scavenging systems might have occurred under saline treatment (Neffati et al., 2011). In our study, a significant lower reducing activity of lavender extracts was detected at NaCl concentrations >50 mM and this paralleled the lower total phenolic amount, being in accordance with findings by Neffati et al. (2011) in coriander.

Mineral foliar application in general did not change the salinity effects (negative trend as salinity increased) on total phenols and antioxidative activity, with some exceptions. More specifically, the positive effects of minerals in antioxidant activity were noticed in low salinity (25 mM NaCl) levels as K and Zn application increased the FRAP activity. However, this scavenging mechanism was not detected in higher (>50 mM NaCl) saline levels, probably due to the plant's lost ability to scavenge ROS (detoxification). Interestingly, the Si application seems to enhance the scavenging mechanism in control treatments, without saline, as both phenolics and FRAP activity significantly increased under the Si foliar treatment, indicating Si induced stress.

Differences in antioxidant enzyme activation could be related to stress intensity (Sgherri and Navari-Izzo, 1995) which depends on the kind of salts and treatment duration (Tarchoune et al., 2010) as well as the species' salt tolerance. In clary sage, the accumulation of phenolics at NaCl concentrations of 25 and 50 mM suggests a relative tolerance of this species to salinity in these two saline concentrations. At a NaCl concentration of 75 mM, there is lower efficiency to eliminate ROS due to imbalance between ROS and antioxidants formation leading to the installation of the oxidative stress (Taarit et al., 2012).

Plants' scavenging ROS capacity is directly related with the activity/content of the antioxidant enzymes (SOD, APX, GR, and CAT) which increase under stress conditions (Foyer and Noctor, 2011). H_2_O_2_ can be removed through the ascorbate-glutathione cycle AsA-GSH, whereas APX and SOD are the key enzymes in this cycle (Pasternak et al., 2005). Salt stress is related to the increased generation of H_2_O_2_ responsible for lipid peroxidation in the absence of any protective mechanism (Xiong and Zhu, 2002). In the present study, the APX enzyme activity increased significantly at NaCl concentrations >50 mM while the opposite occurred for SOD activity. Proline and POD activity also increased at a NaCl concentration of 100 mM. Plants grown under salinity demonstrated lower CAT enzymatic activity. Indeed, NaCl did not significantly change the levels of H_2_O_2_, possibly because of triggered APX activity. Similar findings were reported in basil grown under salinity conditions (Tarchoune et al., 2010).

In plants under salt stress, metabolic shifts to the pentose phosphate pathway lead to an increase in proline synthesis and to a decrease in proline degradation, resulting in higher levels of proline that ultimately enhances the level of erythrose-4-phosphate available to the shikimic acid pathway (Al-Amier and Craker, 2007). High accumulation of proline in leaves is an important adaptive mechanism of salt tolerance as proline is considered to be a source of energy, carbon and nitrogen for the recovering tissues. Proline acts as an osmolyte and reduces the osmotic potential, thus reducing toxic ion uptake (Hare et al., 1998). Similarly to our results, proline content increased in sage and wheat plant grown under saline conditions (Hendawy and Khalid, 2005; Annunziata et al., 2017). These findings are in accordance with those obtained by Woodrow et al. (2017) since they indicated that proline is involved in osmotic adjustment and ROS scavenging. In saline conditions, rosemary plants accumulated Na^+^ to maintain leaf turgor, although they need synthesis of organic solute, especially proline. The accumulation of proline and Na^+^ is a mechanism used for maintaining turgor and reducing the adverse effect of salt stress (Bandeh-Hagh et al., 2008). Proline may act as a radical scavenger and protects cells against salt-induced oxidative stress (Hong et al., 2000). Since total phenolic content and antioxidant activity of salt stressed plants reduced, proline content significantly increased probably acting as a radical scavenger against salt stress.

MDA is used as a marker for evaluation of lipid peroxidation which increases in saline stress conditions. In the presence of oxidative stress more lipid peroxidation products are formed due to cell damage. The increase in lipid peroxidation may be due to the incapability of antioxidants to scavenge ROS results from salt stress, which is not the case for our study. For that reason, MDA was almost unchanged in saline and non-saline treatments. Low antioxidant non enzymatic activity could probably not produce additional hydrogen peroxide from scavenging ROS, so there is no increased H_2_O_2_ to cause high lipid peroxidation. But even if the case was of increased H_2_O_2_, APX activity under high salinity would raise, so it could eliminate any additional H_2_O_2_ coming from another scavenging machine. Several studies found a positive correlation between salt stress and the APX activity (Jahnke and White, 2003; Mehr et al., 2012). In the present study, by increasing NaCl concentration from 0 to 100 mM, POD activity was increased between 34 and 120% in leaves, being in accordance with previous studies (Venkatesan and Chellappan, 1999).

The increased enzymes antioxidant activity in the current study suggests that in both cases the activation of an efficient free radical scavenging system could have minimized the negative effects of a general peroxidation, thus contributing to the maintenance of membrane structure and integrity. Plant tolerance to salinity is related to unchanged general peroxidation level and cell membrane stability (Pérez-López et al., 2009). Therefore, salt tolerance is correlated with the stimulation of antioxidant enzymes and their enhanced ability to scavenge active oxygen species (Tarchoune et al., 2010). This seems to be the most enlightening explanation for the findings of the present study.

The K foliar application increased APX and POD antioxidant activities and proline content, as a result of the increased H_2_O_2_ production in plants grown at NaCl concentrations >50 mM. However, CAT activity was reduced in plants grown under salinity. The increased proline accumulation after K spray at higher salinity concentrations, compared to the unsprayed samples, may be the cause of the increase in H_2_O_2_ content. That could justify the augmentation in enzyme activity of APX and POD after K application as well as SOD activity at levels of samples with 0 mM NaCl. The same trend in enzyme activity after K application was also noticed by Umar et al. (2011). Almost similar results to K application, were found for Zn application (except that H_2_O_2_ production did not vary among salinity levels) and for Si foliar application.

The application of Si in 100 mM NaCl-treated plants alleviated the saline induced stress by means of proline content decrease. Similar to our findings, the NaCl application significantly increased free proline contents in soybean, while, under Si treatment, proline considerably decreased. Earlier reports suggested that silicate partly offsets the adverse impact of NaCl stress, as silicate application increased tomato and soybean tolerance to salinity by raising SOD and CAT activities (as they were increased in this study), chlorophyll content and photochemical efficiency of PSII (Al-Aghabary et al., 2004; Lee et al., 2010). The impact of Si was also noticed via APX activity, where even though it appeared higher in high salinity, activity levels were significantly lower compared to the unsprayed saline plants. It seems that Si decreased lipid peroxidation in salt-stressed plants via enhancing antioxidant enzyme activity and non-enzymatic antioxidants. This has recently been confirmed in experiments with cucumber (Zhu et al., 2004) and tomato (Al-Aghabary et al., 2004). Adding Si decreased the plasma membrane permeability (affecting structure, integrity, and functions of plasma membranes by influencing the stress-dependent peroxidation of membrane lipids) of leaf cells and significantly improved the ultra-structure of chloroplasts (Liang et al., 2005).

Our findings showed an increased H_2_O_2_ production in 100 mM NaCl-treated plants following Si application compared to the non-mineral foliar, indicating that Si acted as an additional stress factor on top of the salinity effects or salinity toxification effects were irreversible. Improvement of salt tolerance by Si addition has been reported in barley (Liang et al., 2003) and cucumber (Zhu et al., 2004), and its role in medicinal plants of the Lamiaceae family, such as lavender, needs to be reconsidered as an assumed means of salinity induced stress alleviation.

In general, oxidative stress might occur through decrease of key antioxidative enzymes (i.e., SOD, CAT) as salinity increases; however, oxidative damage was not evident in the present study as neither H_2_O_2_ nor MDA production increased. The lesser degree of membrane damage (as indicated by low MDA content) and the higher activity of APX and POD observed in NaCl-treated lavender indicated that this particular plant species had a high capacity of scavenging ROS generated by salt stress.

### Mineral content and uptake

Salinity caused a decrease in lavender K^+^ content and K^+^/Na^+^ ratio in treated plants. However, Na^+^ content was increased at all salt levels. Under salinity stress, high Na^+^ uptake competes with the uptake of K^+^ and leads to K^+^/Na^+^ ratio and Na^+^ toxicity decrease. Othman et al. (2006) found that K concentration was reduced by increasing salinity, in accordance with our findings. Na^+^ accumulation in salt stressed plants led to low water potential, changed the ions uptake, and reduced leaf expansion, photosynthetic rate, and plant growth (Zaho et al., 2007). Potassium interacts with almost all essential elements and a synergistic role of K with either N or P has previously been noted (Ranade-Malvi, 2011).

Nutrient disturbances under salinity conditions cause a reduction in plant growth by affecting the availability, transport, and partitioning of the nutrients. Salinity may cause nutrient deficiencies or imbalances because of the competition of Na^+^ and Cl^−^ with nutrients such as K^+^, Ca^2+^, and ${\text{NO}}_{3}^{-}$. Several studies indicated that under salinity conditions, there has been an increase in Na and Cl but a decrease in nitrogen, phosphorus, calcium, potassium, and magnesium levels such as in fennel (Abd El-Wahab, 2006), peppermint and lemon verbena (Tabatabaie and Nazari, 2007), and *Matricaria recutita* (Baghalian et al., 2008).

Salt-stressed root growth is restricted by osmotic effects and toxic effects of ions, which reduce nutrient uptake and inhibit the nutrient translocation, especially K^+^. As a result of the similarities in physicochemical properties, Na^+^ could compete with K^+^ for major binding sites in key metabolic processes, including both low-affinity (e.g., non-selective cation channels) and high-affinity (e.g., K^+^ uptake permeases and high-affinity K^+^ transporter) transporters and could also disturb plant metabolism (Marschner, 2012). Plants use both low and high-affinity systems for potassium uptake. Sodium ions have a more damaging effect on the low-affinity system which has low potassium/sodium selectivity. Under sodium stress, it is necessary for plants to operate the more selective high-affinity potassium uptake system in order to maintain adequate potassium nutrition.

Potassium foliar application did not cause great changes in macronutrients and micronutrients. Potassium levels were increased neither in leaves nor in roots after foliar application of K, in a way to alleviate sodium accumulation in plants' tissue, although potassium levels were increased in roots at 0 mM NaCl+K treatment. This maybe imply that higher concentrations of K could be used in order to manage sodium levels, but that, in this case, that was not possible due to leaf damage, after the exogenous application of higher K concentrations, during the trial versions (preliminary trials) of the experiment. No changes in nutrient content after K foliar application were also reported by Akram and Ashraf (2009), in minerals as Na^+^, Ca^+2^, and Mg^+2^, in sunflower.

Zn application had no specific effects on mineral, although potassium content increased in leaves in high salinity after Zn application. Additionally, zinc content in leaves was found to increase up to six times. Weisany et al. (2014) reported the same changes, among others, after Zn application in soybean grown under salinity. Improving zinc nutritional status of plants growing under saline conditions is of great importance for plants to be protected against toxicity, since zinc is ascribed to have a protective role controlling Na uptake (Cakmak and Marschner, 1988).

Sodium content in the leaves was reduced up to 13% in NaCl concentration 100 mM + Si, suggesting that Si suppresses the translocation of Na from the root to the shoot. Silicon application enhanced K/Na selectivity ratio in *Faba bean* thus enhancing pod and shoot yield (Hellal et al., 2012). The exclusion of Na^+^ ions and a higher K/Na ratio in bean plants grown under saline conditions have been confirmed as a substantial selection criterion for salt tolerance (Abd El-Hamid et al., 2010). In the present study, the ratio of K/Na at NaCl concentration 100 mM averaged at 0.48 while in Si foliar under saline condition (100 mM NaCl) averaged at 0.59 (23% increment). However, in non-saline condition, Si antagonizes the potassium role (appr. of a 45% value), as the K/Na in control (non-saline) averaged 11.58 and in non-saline+Si foliar averaged 6.37. The respective Si:Na in control (non-saline) averaged 33.31 and in non-saline+Si foliar averaged 49.28. The beneficial effect of silicon has been related to the prevention of excessive water loss through transpiration (Savant et al., 1999) or with silicate crystals deposition beneath the epidermal cells of leaves and stems (Trenholm et al., 2004), which may reduce water loss through the cuticles (Lee et al., 2010).

### Essential oil yield and constituents

The essential oil yield in aromatic plants may be affected positively or negatively by the salinity levels (Hendawy and Khalid, 2005; Said-Al Ahl and Omer, 2009; Said-Al Ahl and Mahmoud, 2010; Neffati et al., 2011; Taarit et al., 2012) as well as by the type and amount of fertilizers and cultivation practices applied (Chrysargyris et al., 2016b). Lavender EO yield was reduced in plants grown under NaCl concentration of 100 mM compared to plants grown in non-saline or low saline levels. The foliar application of minerals (K, Zn, and Si) counterbalanced the reduced yield which was observed under the high salinity. The findings of the present study are in agreement with Al-Amier and Craker (2007) on marjoram and with Said-Al Ahl and Mahmoud (2010) and Haddanpouraghdam et al. (2011) on basil, who indicated that saline application in high levels reduced EO yield.

The stimulation of EO production under salinity could be due to a higher oil gland density and an increase in the absolute number of glands produced prior to leaf emergence (Charles et al., 1990). Salt stress may also affect the EO accumulation indirectly through its effects on either net assimilation or assimilate partitioning among growth and differentiation processes (Charles et al., 1990). Morales et al. (1993) suggested that an increase in oil content in some of the salt stressed plants might be attributed to the decline of the primary metabolites due to the salinity effects, causing intermediary products to become available for secondary metabolites synthesis. Additionally, reductions in growth and chlorophylls could be expected to reduce the yield of EO due to fewer metabolites being available for conversion into oil (El-Danasoury et al., 2010) as this was demonstrated in our study.

Said-Al Ahl et al. (2009) reported that potassium-humate increases EO contents and yields in *Thuja orientalis* and oregano, respectively. Similarly, Heidari et al. (2014) reported that foliar K application (in KNO_3_ form) can noticeably improve productivity traits, EO yield, and composition of tarragon plant. On the other hand, other researcher's findings showed that application of K fertilizer did not influence the EO contents of patchouli (Singh and Rao, 2009) and rosemary (Singh et al., 2007). Taking under consideration that zinc is involved in photosynthesis and saccharide metabolism, and since CO_2_ and glucose are the most likely sources of carbon utilized in terpene biosynthesis, the role of zinc in influencing EO accumulation seems principally important (Marschner, 2012).

Analyzing the lavender essential oils, 29–31 individual components were identified in saline and/or foliar treatments. The majority of the EO constituents were oxygenated (monoterpenes and sesquiterpenes) compounds ranged from 69.58 to 79.82% and 2.13 to 4.19%, respectively. It has been reported in *L. aguistifolia* that 1.8-cineole, borneol and camphor were the predominant components of leaf volatile oil, while linalool, 1.8-cineole, borneol and camphor were the major components of inflorescence oil (Haddanpouraghdam et al., 2011; Chrysargyris et al., 2016b), being in accordance with the current leaf oil composition, indicating the lavender chemotype (CT) of CT-1.8 cineole. Oil quality decreases with increasing camphor ratios (Biswas et al., 2009) and the NaCl concentration of 25 mM is considered as the most appropriate one regarding the lowest camphor content. Borneol is easily oxidized to the camphor (ketone) and this was evidenced for the NaCl concentrations 0 and 50 mM. Salinity may change the content of several oil constituents by altering biosynthetic processes. Hendawy and Khalid (2005) reported that 2,500 ppm soil salinity increased α-thujone, camphor and 1.8-cineole, but decreased β-thujone compared with the control treatment in *Salvia officinalis*. The application of saline levels ranging from 0 to 100 mM NaCl did not change the 1.8-cineole and D-limonene content, the most abundant constituents in lavender leaf EO. The 1.8-cineole, also known as eucalyptol, due to its pleasant spicy aroma and taste is extensively used in cosmetics, fragrances and flavorings as well as an insecticide and insect repellent (Sfara et al., 2009).

Interestingly, the K foliar application in plants subjected to different salinity levels changed the chemical composition of the lavender EO, and occasionally altered the salinity effects on oil composition. For example, borneol content remained similar among treatments which actually resulted in similar camphor content. Another issue is that K application enhanced the 1.8-cineole content at a NaCl concentration of 100 mM due to the reduced α-terpineol, as a precursor stage of 1.8-cineole. In this direction, K application in high salinity (100 mM NaCl) affected the geranyl diphosphate biosynthesis by altering the formation of D-limonene, α-pinene, β-pinene, and sabinene, by reducing their content, preventing limonene and pinene degradation (Sell, 2003). Similarly, K application in high salinity (100 mM NaCl) affected the geranyl diphosphate biosynthesis by reducing the formation of β-myrcene, and as a consequence delaying the formation of (+)-linalool. K application at NaCl concentrations <50 mM delayed the 1.8-cineole formation and enhanced D-limonene, α-pinene, β-pinene, and β-myrcene biosynthesis.

In the present study, salinity (100 mM NaCl) had negative effects on the biosynthesis and accumulation of α-pinene and β-myrcene, while both Zn and Si foliar applications had influential potential to compensate for the deteriorative effects of salinity depression on these compounds. Coolong et al. (2004) suggested that Zn fertility can influence changes in glucosinolates that may affect related plants flavor or medicinal features. Apparently, moderate salinity levels when combined with raising Zn levels through foliar application had synergistic effects on production of some compounds (Haddanpouraghdam et al., 2011). Misra and Sharma (1991) mentioned that Zn application stimulated menthol concentration in Japanese mint.

Based on our findings, monoterpenes were predominant compared to the sesquiterpenes, indicating the 1-deoxy-D-xylulose-5-phosphate (DXP) pathway activation, which is localized in plastids, compared to the classical mevalonic acid (MVA) pathway—it operates in cytocol and produces precursor for the biosynthesis of sesquiterpenes (which were in low percentages in the present study) and triterpenes, being in accordance with previous findings in lavender (Lane et al., 2010). High yielding lavender varieties (e.g., *Lavandin*; *Lavandula intermedia*) produce lower quality oil. Therefore, plant biomass and EO yield are not the only parameters that should be considered by investigators, although great importance is given to the oil composition, as well.

## Conclusion

Our results have demonstrated that lavender growth/development and EO production may be affected by saline levels, whereas mechanisms for alteration of induced stress are of great importance. High levels of salinity seem to reduce lavender growth and EO yield, and this is directly related to the crop reduced marketability when used as either ornamental or for essential oil production. In general, low-moderate (20–50 mM) salinity maintained the oil composition profile for lavender. The combined foliar application of K and Zn ameliorated the apparent salinity negative effects on fresh biomass produced, EO yield and maintained, to some extent, the antioxidant properties of the saline-stressed plants. Evidently, lavender primary metabolites affected by moderate salinity stress along with K foliar operated in favor of major volatile oil components biosynthesis. Thus, lavender crop has the potential to be expanded and cultivated in semi-saline conditions. Through the current study, it has been shown that Zn and Si application, seems to have a smaller impact on the composition of EO, despite the fact that minerals alleviated salinity induced changes. The efficiency of Zn and Si should be approached in the context of optimum growing conditions and balanced micronutrients availability, while the role of iron and manganese could also be considered in future studies.

## Author contributions

AC, EM, and NT: designing and performing the experiments; AC: GC/MS, ICP-OES analysis; EM and NT: physiological measurements; AC and NT: data analysis and critical discussion of the data; NT and AC: paper preparation; NT: research coordination.

### Conflict of interest statement

The authors declare that the research was conducted in the absence of any commercial or financial relationships that could be construed as a potential conflict of interest.
