# Supplementary material for: Physiological and Biochemical Responses of Lavandula angustifolia to Salinity Under Mineral Foliar Application
Source: Front Plant Sci. 2018 Apr 20;9:489. doi: 10.3389/fpls.2018.00489 (PMC5920160; doi:10.3389/fpls.2018.00489)
Supplement: Supplementary file 1 [file Table1.docx]

**Table S1.** Effect of salinity levels (0-25-50-100 mM NaCl) and foliar applications (no foliar, K, Zn and Si) on the root analysis in lavender grown hydroponically in perlite.

|  |  | **Macronutrients** | | | | |  |
| --- | --- | --- | --- | --- | --- | --- | --- |
| ***Significance*** | **N** | **K** | **P** | **Ca** | **Mg** | **Na** |  |
| ***Salinity (S)*** | * | *** | *** | *** | *** | *** |  |
| ***Foliar (F)*** | *ns* | ** | *ns* | *ns* | *ns* | *ns* |  |
| ***S x F*** | *ns* | *ns* | *ns* | *ns* | *ns* | *ns* |  |
|  |  |  | **Micronutrients** | | | |  |
| ***Significance*** | **Al** | **Fe** | **B** | **Cu** | **Mn** | **Zn** | **Si** |
| ***Salinity (S)*** | *ns* | ** | *** | ** | *** | *** | *** |
| ***Foliar (F)*** | *ns* | *ns* | *ns* | *ns* | *ns* | * | *ns* |
| ***S x F*** | *ns* | *ns* | *ns* | *ns* | *ns* | *ns* | *ns* |

ns, *, **, and *** indicate non-significant or significant differences at *P*< 5%, 1% and 0.1%, respectively, following two-way ANOVA.
