# Supplementary material for: Physiological and Biochemical Responses of Lavandula angustifolia to Salinity Under Mineral Foliar Application
Source: Front Plant Sci. 2018 Apr 20;9:489. doi: 10.3389/fpls.2018.00489 (PMC5920160; doi:10.3389/fpls.2018.00489)
Supplement: Supplementary file 2 [file Table2.docx]

**Table S2.** Chemical composition (%) of essential oils of lavender plants grown hydroponically under different salinity levels.

|  |  | **Salinity NaCl** | | | | |
| --- | --- | --- | --- | --- | --- | --- |
| **Compound** | **RI** | **0mM** | **25mM** | **50mM** | **100mM** |  |
| α-Pinene | 933 | **4.45±0.15a** | **4.33±0.17a** | **4.26±0.10ab** | **3.86±0.02b** |  |
| Camphene | 948 | 0.41±0.01ab | 0.39±0.01b | 0.48±0.03a | 0.42±0.00ab |  |
| Sabinene | 973 | **2.49±0.08a** | **2.49±0.09a** | **2.50±0.14a** | **2.28±0.02a** |  |
| β-Pinene | 977 | **7.38±0.24a** | **7.42±0.19a** | **7.45±0.26a** | **6.76±0.02a** |  |
| β-Myrcene | 991 | **2.64±0.13ab** | **2.98±0.07a** | **2.73±0.24ab** | **2.40±0.02b** |  |
| α-Phellandrene | 1008 | 0.06±0.00ab | 0.08±0.00a | 0.06±0.01ab | 0.05±0.00b |  |
| α-Terpinene | 1017 | 0.06±0.00b | 0.10±0.00a | 0.11±0.01a | 0.10±0.00a |  |
| o-Cymene | 1024 | 0.05±0.00b | 0.05±0.00b | 0.10±0.02a | 0.10±0.00a |  |
| D-Limonene | 1028 | **7.50±0.37a** | **9.12±0.31a** | **8.67±0.89a** | **7.39±0.06a** |  |
| 1.8-Cineole | 1031 | **57.20±1.07a** | **54.64±1.35a** | **54.12±2.19a** | **56.38±0.47a** |  |
| *cis*-Ocimene | 1035 | 0.19±0.01b | 0.35±0.03a | 0.25±0.03b | 0.17±0.00b |  |
| γ-Terpinene | 1058 | 0.14±0.01b | 0.21±0.01a | 0.24±0.03a | 0.26±0.00a |  |
| *cis*-Sabinenehydrate | 1067 | 0.52±0.04a | 0.33±0.03b | 0.27±0.02b | 0.26±0.01b |  |
| p-Mentha-2,4(8)-diene | 1089 | 0.18±0.01b | 0.23±0.00a | 0.21±0.01ab | 0.17±0.00b |  |
| Linolool | 1100 | 0.21±0.00a | 0.22±0.01a | 0.17±0.01b | 0.16±0.00b |  |
| *trans-*Pinocarveol | 1139 | 0.10±0.01b | 0.08±0.00b | 0.13±0.02b | 0.19±0.00a |  |
| Camphor | 1145 | **5.67±0.09a** | **4.97±0.04b** | **5.63±0.32a** | **5.76±0.05a** |  |
| Pinocarvone | 1163 | 0.14±0.01b | 0.14±0.01b | 0.25±0.04a | 0.32±0.01a |  |
| Borneol | 1166 | **3.07±0.13b** | **3.43±0.18b** | **3.42±0.14b** | **4.25±0.05a** |  |
| Terpinen-4-ol | 1178 | 0.20±0.00b | 0.30±0.04ab | 0.25±0.03ab | 0.35±0.01a |  |
| Cryptone | 1187 | 0.09±0.01ab | 0.07±0.02b | 0.09±0.00ab | 0.12±0.00a |  |
| α-Terpineol | 1191 | **2.77±0.08a** | **2.77±0.13a** | **2.32±0.16b** | **2.29±0.03b** |  |
| Myrtenal | 1197 | 0.29±0.03b | 0.26±0.03b | 0.51±0.09a | 0.63±0.01a |  |
| Cumic aldehyde | 1241 | 0.06±0.01b | 0.07±0.01b | 0.16±0.03a | 0.20±0.00a |  |
| β-Caryophyllene | 1424 | 0.10±0.01a | 0.13±0.02a | 0.15±0.03a | 0.12±0.01a |  |
| α-Alaskene | 1526 | 0.71±0.06a | 0.71±0.07a | 0.91±0.15a | 0.81±0.03a |  |
| tau-Cadinol | 1642 | 0.27±0.02b | 0.31±0.04ab | 0.40±0.06ab | 0.044±0.01a |  |
| α-Bisabolol | 1685 | **1.15±0.10a** | **1.25±0.16a** | **1.51±0.27a** | **1.56±0.05a** |  |
| *cis*-Lanceol | 1749 | **1.20±0.17a** | **1.71±0.21a** | **1.68±0.39a** | **1.38±0.05a** |  |
|  |  |  |  |  |  |  |
| Monoterpenes hydrocarbons | | 22.95±0.90ab | 24.80±0.78a | 24.36±1.38ab | 21.59±0.10b |  |
| Sesquiterpenes hydrocarbons | | 0.82±0.08a | 0.85±0.08a | 1.06±0.18a | 0.93±0.04a |  |
| Oxygenated monoterpenes | | 72.90±1.32a | 70.23±1.37a | 70.00±2.49a | 73.26±0.25a |  |
| Oxygenated sesquiterpenes | | 2.62±0.29a | 3.27±0.41a | 3.60±0.72a | 3.38±0.13a |  |
| Others |  | 0.09±0.01ab | 0.07±0.01b | 0.09±0.00ab | 0.12±0.00a |  |
| **Total** |  | 99.39±0.06a | 99.23±0.07a | 99.13±0.20a | 99.26±0.05a |  |

Values (n=3) in rows followed by the same letter are not significantly different, *P*<0.05.

**Table S3.** Chemical composition (%) of essential oils of lavender plants grown hydroponically under different salinity levels with K foliar application.

|  |  | **Salinity NaCl +Foliar K** | | | |  |
| --- | --- | --- | --- | --- | --- | --- |
| **Compound** | **RI** | **0mM** | **25mM** | **50mM** | **100mM** | |
| α-Pinene | 933 | **4.41±0.02a** | **4.05±0.28a** | **3.91±0.19ab** | **3.30±0.04b** | |
| Camphene | 948 | 0.39±0.02a | 0.38±0.01a | 0.42±0.03a | 0.38±0.00a | |
| Sabinene | 973 | **2.41±0.17a** | **2.42±0.09a** | **2.40±0.10a** | **1.79±0.01b** | |
| β-Pinene | 977 | **7.11±0.42a** | **7.09±0.33a** | **6.96±0.30a** | **5.50±0.03b** | |
| β-Myrcene | 991 | **2.73±0.19a** | **2.86±0.13a** | **2.67±0.12a** | **1.72±0.01b** | |
| α-Phellandrene | 1008 | 0.07±0.00a | 0.07±0.00a | 0.06±0.00a | 0.00±0.00b | |
| α-Terpinene | 1017 | 0.08±0.00a | 0.09±0.01a | 0.09±0.01a | 0.12±0.00a | |
| D-Limonene | 1028 | **7.36±0.58a** | **8.35±0.44a** | **8.45±0.59a** | **5.17±0.09b** | |
| 1.8-Cineole | 1031 | **53.73±2.14b** | **52.22±0.81b** | **53.21±0.96b** | **62.53±0.23a** | |
| *cis*-Ocimene | 1035 | 0.23±0.00a | 0.27±0.00a | 0.27±0.05a | 0.08±0.00b | |
| γ-Terpinene | 1058 | 0.18±0.01a | 0.19±0.01a | 0.21±0.04a | 0.26±0.00a | |
| *cis*-Sabinenehydrate | 1067 | 0.53±0.07a | 0.50±0.03a | 0.42±0.09ab | 0.26±0.00b | |
| p-Mentha-2,4(8)-diene | 1089 | 0.23±0.02a | 0.24±0.01a | 0.19±0.00a | 0.12±0.00b | |
| Linalool | 1100 | 0.27±0.01a | 0.27±0.01a | 0.22±0.01b | 0.14±0.00c | |
| *trans-*Pinocarveol | 1139 | 0.13±0.02b | 0.10±0.02b | 0.17±0.02b | 0.30±0.01a | |
| Camphor | 1145 | **5.09±0.37a** | **4.70±0.58a** | **5.15±0.36a** | **5.37±0.07a** | |
| Pinocarvone | 1163 | 0.19±0.03bc | 0.16±0.03c | 0.26±0.01ab | 0.31±0.01a | |
| Borneol | 1166 | **4.72±0.32a** | **5.14±0.95a** | **4.81±0.84a** | **5.41±0.05a** | |
| Terpinen-4-ol | 1178 | 0.32±0.00b | 0.34±0.01b | 0.29±0.05b | 0.46±0.00a | |
| Cryptone | 1187 | 0.11±0.01b | 0.11±0.00b | 0.14±0.01b | 0.20±0.00a | |
| α-Terpineol | 1191 | **3.25±0.18ab** | **3.41±0.21a** | **2.69±0.25bc** | **2.41±0.05c** | |
| Myrtenal | 1197 | 0.39±0.06bc | 0.34±0.08c | 0.54±0.02ab | 0.68±0.01a | |
| Cumic aldehyde | 1241 | 0.09±0.01b | 0.08±0.02b | 0.18±0.01a | 0.18±0.00a | |
| β-Caryophyllene | 1424 | 0.18±0.03b | 0.21±0.00a | 0.17±0.02a | 0.04±0.00a | |
| α-Alaskene | 1526 | 0.83±0.08a | 0.92±0.05a | 0.93±0.02a | 0.44±0.01b | |
| Caryophyllene oxide | 1587 | 0.01±0.01bc | 0.00±0.00c | 0.05±0.02ab | 0.07±0.00a | |
| tau-Cadinol | 1642 | 0.43±0.04a | 0.43±0.02a | 0.46±0.01a | 0.28±0.00b | |
| α-Bisabolol | 1685 | **1.67±0.13a** | **1.69±0.03a** | **1.69±0.01a** | **1.03±0.03b** | |
| Muurola-5-en-4-one | 1689 | 0.03±0.00b | 0.07±0.01b | 0.11±0.01a | 0.13±0.00a | |
| *cis*-Lanceol | 1749 | **1.84±0.23a** | **2.07±0.06a** | **1.87±0.07a** | **0.73±0.01b** | |
|  | |  |  |  |  | |
| Monoterpenes hydrocarbons | | 22.43±1.58a | 23.18±1.16a | 22.98±1.25a | 16.74±0.10b | |
| Sesquiterpenes hydrocarbons | | 1.00±0.11a | 1.13±0.06a | 1.11±0.05a | 0.48±0.01b | |
| Oxygenated monoterpenes | | 71.47±2.11b | 70.17±1.04b | 70.62±1.35b | 79.82±0.09a | |
| Oxygenated sesquiterpenes | | 3.95±0.38a | 4.19±0.01a | 4.08±0.09a | 2.13±0.05b | |
| Others |  | 0.13±0.02c | 0.16±0.03c | 0.25±0.01b | 0.33±0.00a | |
| **Total** |  | 99.00±0.17a | 98.86±0.08a | 99.06±0.08a | 99.52±0.02a | |

Values (n=3) in rows followed by the same letter are not significantly different, *P*<0.05.

**Table S4.** Chemical composition (%) of essential oils of lavender plants grown hydroponically under different salinity levels with Zn foliar application.

|  |  | **Salinity NaCl +Foliar Zn** | | | | |
| --- | --- | --- | --- | --- | --- | --- |
| **Compound** | **RI** | **0mM** | **25mM** | **50mM** | **100mM** |  |
| α-Pinene | 933 | **4.07±0.32a** | **3.98±0.16a** | **3.98±0.06a** | **4.01±0.32a** |  |
| Camphene | 948 | 0.40±0.02a | 0.38±0.01a | 0.38±0.00a | 0.44±0.04a |  |
| Sabinene | 973 | **2.33±0.18a** | **2.39±0.10a** | **2.27±0.02a** | **2.42±0.10a** |  |
| β-Pinene | 977 | **7.04±0.44a** | **7.13±0.22a** | **6.74±0.05a** | **7.10±0.34a** |  |
| β-Myrcene | 991 | **2.47±0.19a** | **2.65±0.13a** | **2.44±0.01a** | **2.50±0.05a** |  |
| α-Phellandrene | 1008 | 0.03±0.02a | 0.04±0.02a | 0.03±0.01a | 0.05±0.00a |  |
| α-Terpinene | 1017 | 0.07±0.00b | 0.07±0.00b | 0.08±0.00ab | 0.11±0.02a |  |
| o-Cymene | 1024 | 0.07±0.01b | 0.06±0.00b | 0.06±0.00b | 0.10±0.00a |  |
| D-Limonene | 1028 | **7.37±0.54a** | **7.98±0.65a** | **7.14±0.14a** | **7.91±0.16a** |  |
| 1.8-Cineole | 1031 | **55.55±1.90a** | **54.15±1.04a** | **56.63±0.86a** | **54.90±0.40a** |  |
| *cis*-Ocimene | 1035 | 0.14±0.00b | 0.23±0.02a | 0.19±0.01a | 0.20±0.00a |  |
| γ-Terpinene | 1058 | 0.17±0.01b | 0.16±0.01b | 0.18±0.01b | 0.20±0.00a |  |
| *cis*-Sabinenehydrate | 1067 | 0.49±0.04a | 0.47±0.01a | 0.39±0.05a | 0.32±0.07a |  |
| p-Mentha-2,4(8)-diene | 1089 | 0.18±0.01a | 0.18±0.01a | 0.16±0.00a | 0.17±0.00a |  |
| Linalool | 1100 | 0.22±0.02a | 0.22±0.01a | 0.20±0.02a | 0.17±0.01a |  |
| *trans-*Pinocarveol | 1139 | 0.13±0.02ab | 0.11±0.00b | 0.15±0.01ab | 0.17±0.01a |  |
| Camphor | 1145 | **5.75±0.19a** | **5.14±0.46a** | **5.16±0.17a** | **5.53±0.15a** |  |
| Pinocarvone | 1163 | 0.19±0.02b | 0.16±0.00b | 0.19±0.01b | 0.28±0.00a |  |
| Borneol | 1166 | **3.98±0.21a** | **4.69±0.68a** | **4.65±0.38a** | **4.10±0.18a** |  |
| Terpinen-4-ol | 1178 | 0.27±0.03a | 0.27±0.04a | 0.36±0.02a | 0.31±0.02a |  |
| Cryptone | 1187 | 0.13±0.01b | 0.13±0.01b | 0.17±0.01a | 0.11±0.00b |  |
| α-Terpineol | 1191 | **2.93±0.21a** | **3.06±0.24a** | **3.07±0.13a** | **2.49±0.10a** |  |
| Myrtenal | 1197 | 0.42±0.05b | 0.36±0.02b | 0.41±0.03b | 0.60±0.02a |  |
| Cumic aldehyde | 1241 | 0.11±0.01b | 0.011±0.00b | 0.11±0.00b | 0.19±0.00a |  |
| β-Caryophyllene | 1424 | 0.13±0.03a | 0.15±0.01a | 0.11±0.02a | 0.11±0.01a |  |
| α-Alaskene | 1526 | 0.87±0.07a | 0.89±0.07a | 0.73±0.04a | 0.87±0.03a |  |
| Caryophyllene oxide | 1587 | 0.03±0.01b | 0.01±0.01b | 0.01±0.01b | 0.08±0.00a |  |
| tau-Cadinol | 1642 | 0.42±0.01b | 0.45±0.00ab | 0.40±0.02b | 0.48±0.00a |  |
| α-Bisabolol | 1685 | **1.71±0.04a** | **1.78±0.00a** | **1.52±0.08b** | **1.78±0.04a** |  |
| Muurola-5-en-4-one | 1689 | 0.03±0.01b | 0.01±0.01b | 0.04±0.02b | 0.15±0.01a |  |
| *cis*-Lanceol | 1749 | **1.51±0.19a** | **1.78±0.08a** | **1.41±0.13a** | **1.45±0.05a** |  |
|  |  |  |  |  |  |  |
| Monoterpenes hydrocarbons | | 21.90±1.52a | 22.64±1.18a | 21.24±0.24a | 22.82±0.93a |  |
| Sesquiterpenes hydrocarbons | | 1.00±0.10a | 1.04±0.07a | 0.85±0.06a | 0.98±0.04a |  |
| Oxygenated monoterpenes | | 72.55±1.95a | 71.43±1.33a | 73.80±0.44a | 71.59±0.77a |  |
| Oxygenated sesquiterpenes | | 3.71±0.21ab | 4.05±0.06a | 3.40±0.25b | 3.96±0.10ab |  |
| Others |  | 0.16±0.02b | 0.14±0.02b | 0.21±0.03ab | 0.26±0.01a |  |
| **Total** |  | 99.35±0.16a | 99.31±0.08a | 99.52±0.04a | 99.62±0.04a |  |

Values (n=3) in rows followed by the same letter are not significantly different, *P*<0.05.

**Table S5.** Chemical composition (%) of essential oils of lavender plants grown hydroponically under different salinity levels with Si foliar application.

|  |  | **Salinity NaCl +Foliar Si** | | | | |
| --- | --- | --- | --- | --- | --- | --- |
| **Compound** | **RI** | **0mM** | **25mM** | **50mM** | **100mM** |  |
| α-Pinene | 933 | **4.00±0.25a** | **3.89±0.13a** | **4.21±0.25a** | **4.47±0.00a** |  |
| Camphene | 948 | 0.39±0.01b | 0.38±0.01b | 0.44±0.00a | 0.46±0.01a |  |
| Sabinene | 973 | **2.39±0.15a** | **2.26±0.08a** | **2.48±0.06a** | **2.62±0.00a** |  |
| β-Pinene | 977 | **7.15±0.41a** | **6.81±0.25a** | **7.27±0.18a** | **7.51±0.03a** |  |
| β-Myrcene | 991 | **2.59±0.22a** | **2.53±0.16a** | **2.77±0.06a** | **2.65±0.03a** |  |
| α-Phellandrene | 1008 | 0.05±0.01a | 0.05±0.00a | 0.04±0.02a | 0.02±0.02a |  |
| α-Terpinene | 1017 | 0.06±0.00a | 0.05±0.00b | 0.07±0.00a | 0.07±0.00a |  |
| o-Cymene | 1024 | 0.06±0.01a | 0.06±0.01a | 0.08±0.02a | 0.09±0.00a |  |
| D-Limonene | 1028 | **7.43±0.72b** | **7.86±0.30ab** | **9.23±0.36a** | **8.30±0.26ab** |  |
| 1.8-Cineole | 1031 | **56.74±1.79a** | **56.93±0.39a** | **53.06±0.76a** | **55.05±0.53a** |  |
| *cis*-Ocimene | 1035 | 0.22±0.04a | 0.23±0.03a | 0.25±0.01a | 0.18±0.00a |  |
| γ-Terpinene | 1058 | 0.15±0.01a | 0.11±0.00b | 0.16±0.00a | 0.17±0.00a |  |
| *cis*-Sabinenehydrate | 1067 | 0.56±0.00a | 0.60±0.03a | 0.47±0.02b | 0.40±0.1b |  |
| p-Mentha-2,4(8)-diene | 1089 | 0.18±0.02a | 0.16±0.01a | 0.19±0.00a | 0.17±0.00a |  |
| Linalool | 1100 | 0.22±0.01a | 0.21±0.01a | 0.19±0.00a | 0.15±0.00b |  |
| *trans-*Pinocarveol | 1139 | 0.12±0.02a | 0.11±0.02a | 0.13±0.02a | 0.12±0.01a |  |
| Camphor | 1145 | **5.42±0.39a** | **5.03±0.39a** | **5.58±0.09a** | **5.21±0.03a** |  |
| Pinocarvone | 1163 | 0.2±0.02a | 0.19±0.02a | 0.23±0.04a | 0.25±0.01a |  |
| Borneol | 1166 | **3.88±0.35a** | **4.64±0.56a** | **3.81±0.19a** | **3.45±0.06a** |  |
| Terpinen-4-ol | 1178 | 0.19±0.01a | 0.17±0.00a | 0.19±0.01a | 0.17±0.00a |  |
| Cryptone | 1187 | 0.09±0.01a | 0.09±0.01a | 0.10±0.00a | 0.03±0.03b |  |
| α-Terpineol | 1191 | **2.82±0.07a** | **2.77±0.09a** | **2.48±0.20ab** | **2.11±0.04b** |  |
| Myrtenal | 1197 | 0.39±0.05a | 0.36±0.09a | 0.46±0.10a | 0.47±0.01a |  |
| Cumic aldehyde | 1241 | 0.11±0.01a | 0.10±0.01a | 0.16±0.04a | 0.15±0.00a |  |
| β-caryophyllene | 1424 | 0.09±0.01ab | 0.08±0.00b | 0.17±0.03a | 0.14±0.01ab |  |
| α-Alaskene | 1526 | 0.70±0.07a | 0.64±0.03a | 0.90±0.15a | 0.94±0.01a |  |
| Caryophyllene oxide | 1587 | 0.00±0.00a | 0.00±0.00a | 0.07±0.03a | 0.07±0.00a |  |
| tau-Cadinol | 1642 | 0.34±0.04a | 0.29±0.01a | 0.44±0.06a | 0.45±0.01a |  |
| α-Bisabolol | 1685 | **1.37±0.13a** | **1.30±0.04a** | **1.69±0.19a** | **1.73±0.04a** |  |
| Muurola-5-en-4-one | 1689 | 0.02±0.01ab | 0.00±0.00b | 0.09±0.05ab | 0.12±0.01b |  |
| *cis*-Lanceol | 1749 | **1.38±0.17a** | **1.42±0.07a** | **1.75±0.09a** | **1.57±0.04a** |  |
|  |  |  |  |  |  |  |
| Monoterpenes hydrocarbons | | 22.08±1.63a | 21.89±0.78a | 24.45±0.22a | 24.08±0.25a |  |
| Sesquiterpenes hydrocarbons | | 0.80±0.09a | 0.73±0.03a | 1.07±0.19a | 1.08±0.02a |  |
| Oxygenated monoterpenes | | 73.27±2.02a | 73.66±0.76a | 69.58±0.65a | 70.20±0.32a |  |
| Oxygenated sesquiterpenes | | 3.10±0.34a | 3.01±0.04a | 3.96±0.38a | 3.82±0.10a |  |
| Others |  | 0.11±0.02a | 0.09±0.01a | 0.19±0.06a | 0.15±0.02a |  |
| **Total** |  | 99.38±0.07a | 99.40±0.00a | 99.26±0.10a | 99.35±0.03a |  |

Values (n=3) in rows followed by the same letter are not significantly different, *P*<0.05.
