# Supplementary figures and images for: Physiological and Biochemical Responses of Lavandula angustifolia to Salinity Under Mineral Foliar Application
Source: Front Plant Sci. 2018 Apr 20;9:489. doi: 10.3389/fpls.2018.00489 (PMC5920160; doi:10.3389/fpls.2018.00489)

**
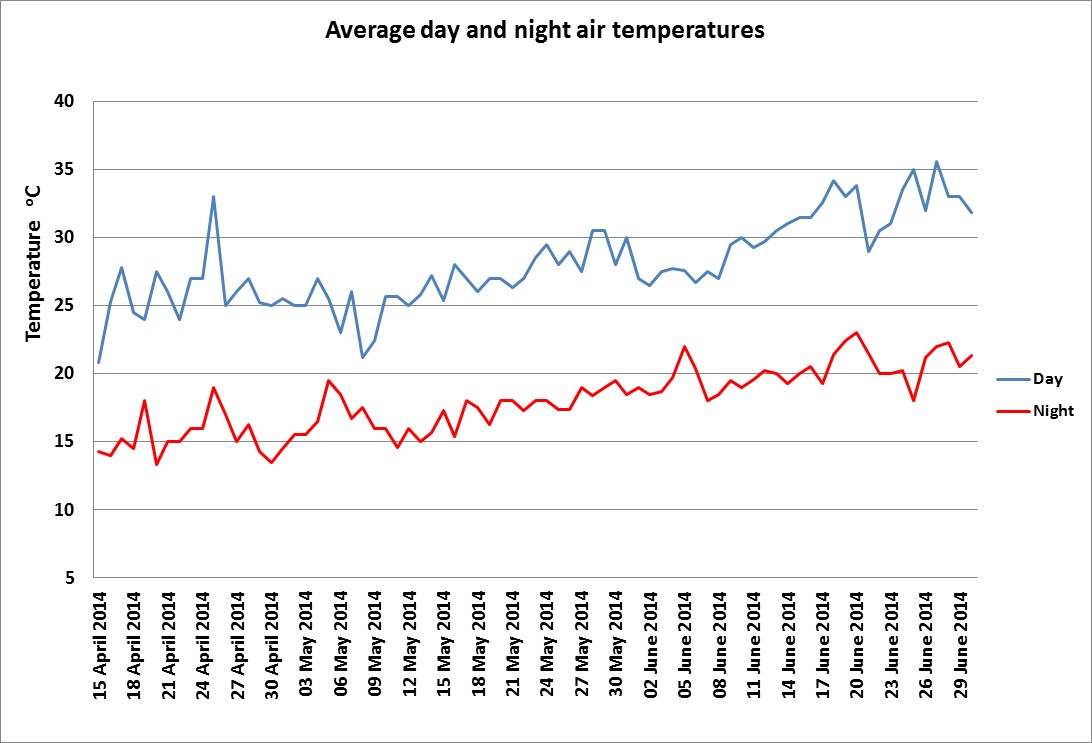
**

**Figure S1.** Daily average day and night temperature during the experiment period.

Supplement: Supplementary file 3 [file DataSheet1.docx]
