# Supplementary material for: Physiological and Biochemical Responses of Lavandula angustifolia to Salinity Under Mineral Foliar Application
Source: Front Plant Sci. 2018 Apr 20;9:489. doi: 10.3389/fpls.2018.00489 (PMC5920160; doi:10.3389/fpls.2018.00489)
Supplement: Supplementary file 4 [file DataSheet2.DOCX]

| **No foliar** | **+K** | **+Zn** | **+Si** |
| --- | --- | --- | --- |

**
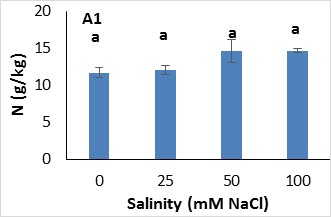

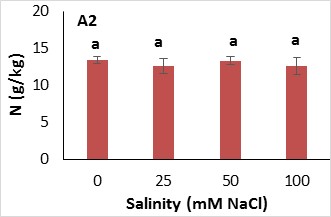

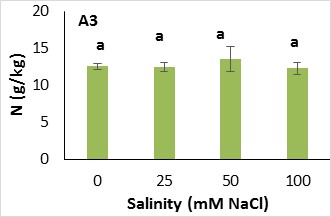

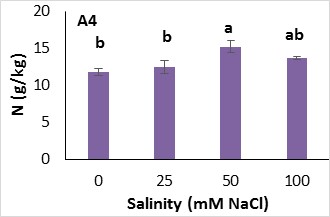
**

**
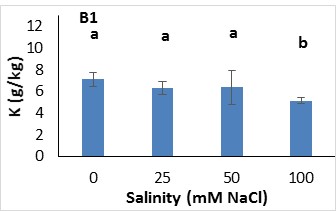

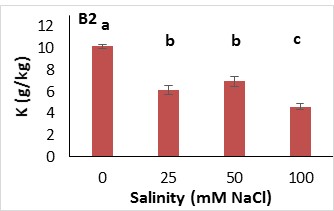

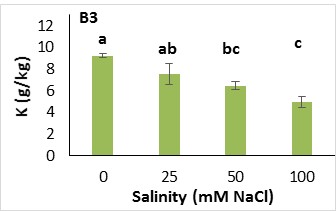

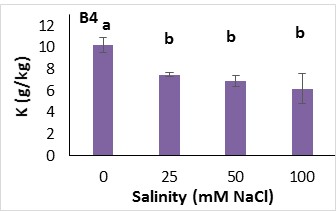
**

**
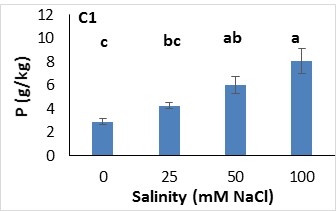

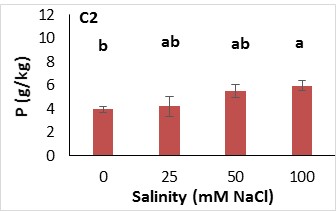

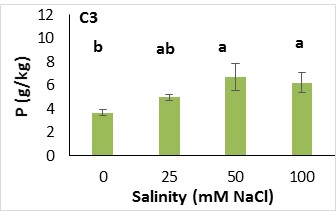

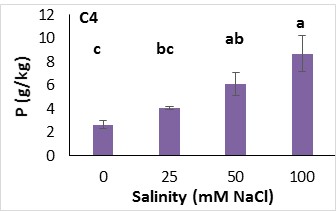
**

**
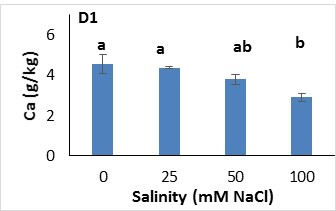

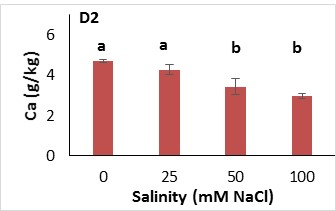

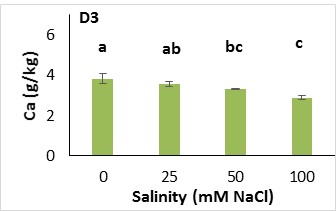

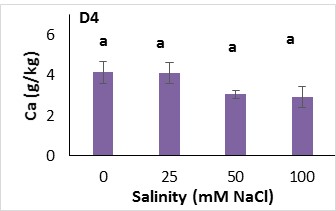
**

**
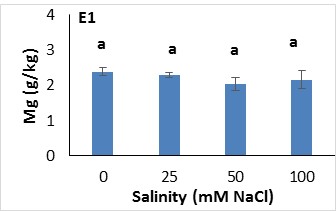

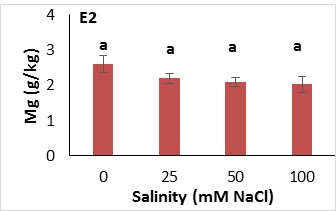

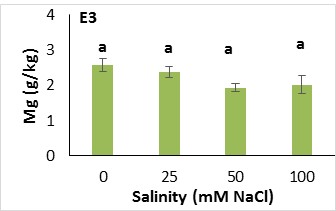

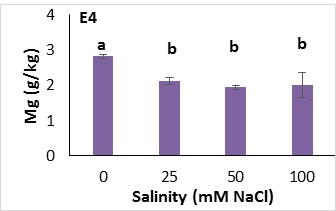
**

**
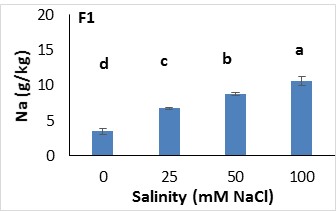

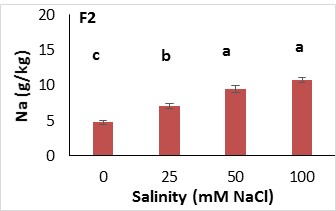

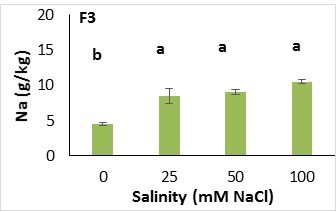

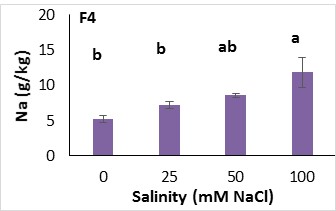
**

**
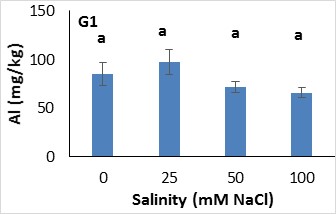

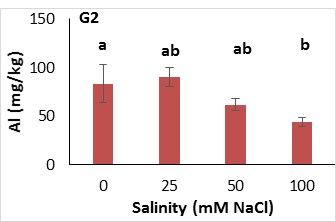

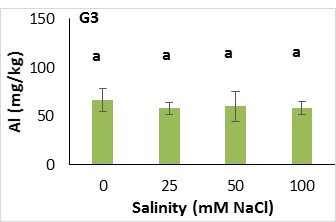

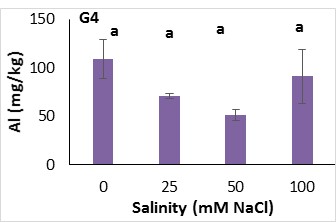
**

**
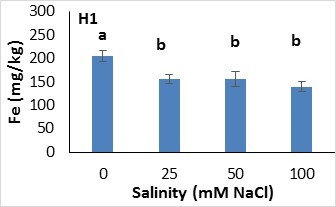

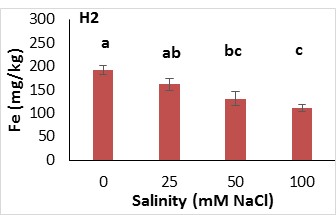

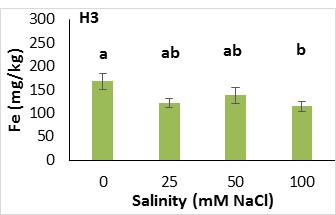

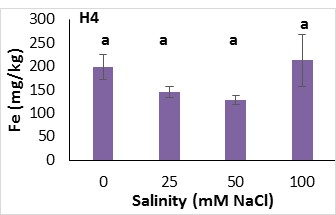
**

**
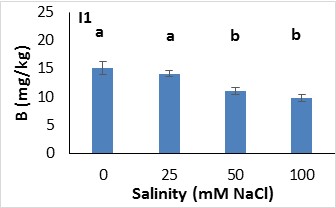

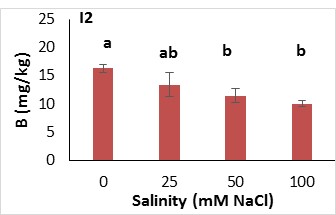

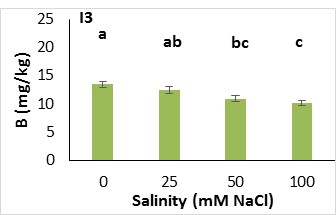

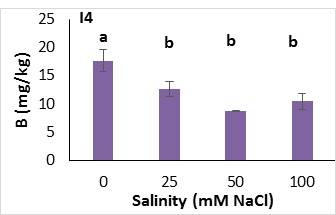
**

**
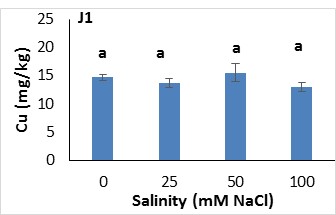

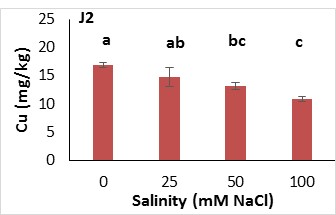

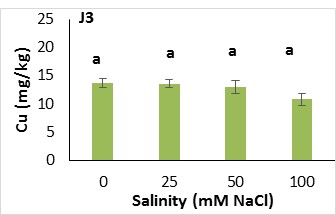

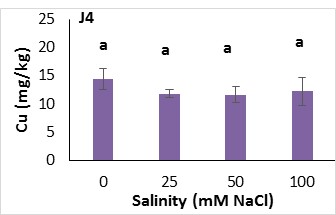
**

**
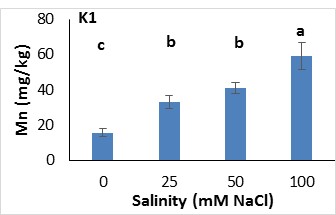

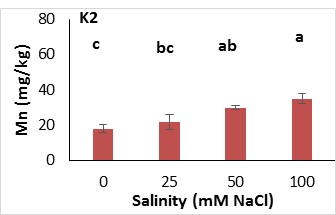

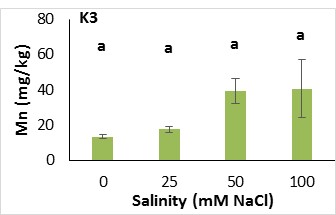

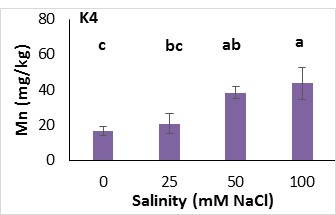
**

**
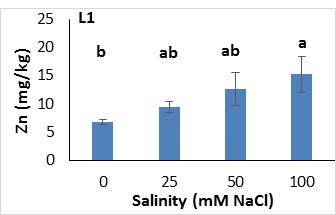

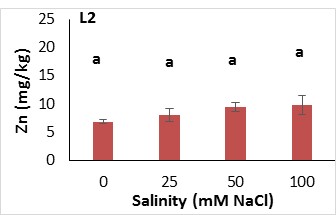

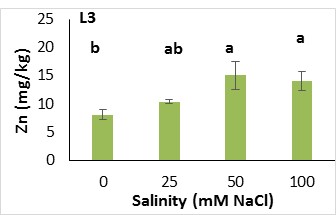

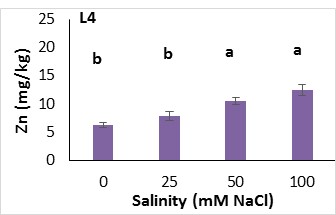
**

**
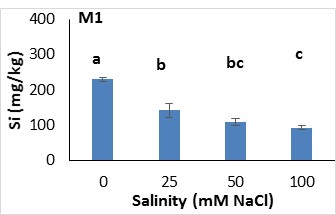

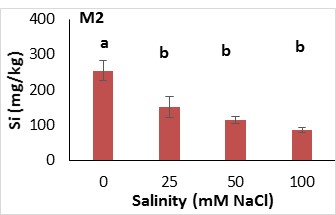

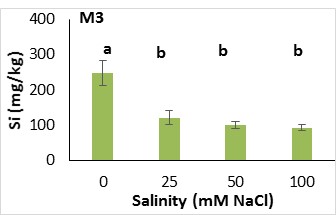

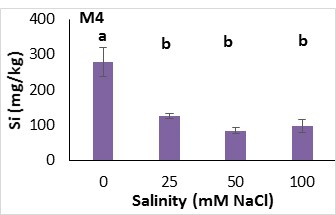
**

**Figure S2:** Lavender root analysis of plants grown hydroponically in perlite under different salinity levels (0-25-50-100 mM NaCl) and foliar applications (no foliar, K, Zn and Si). Sub-figures **(A–M)** referring to different macro- and micronutrients and numbering **(1–4)** referring to the no foliar, K, Zn and Si, respectively. Significant differences (*P*<0.05) among treatments are indicated by different letters. Error bars show SE (n=3).
