# Supplementary material for: Physiological and Biochemical Responses of Lavandula angustifolia to Salinity Under Mineral Foliar Application
Source: Front Plant Sci. 2018 Apr 20;9:489. doi: 10.3389/fpls.2018.00489 (PMC5920160; doi:10.3389/fpls.2018.00489)
Supplement: Supplementary file 5 [file Presentation1.pdf]

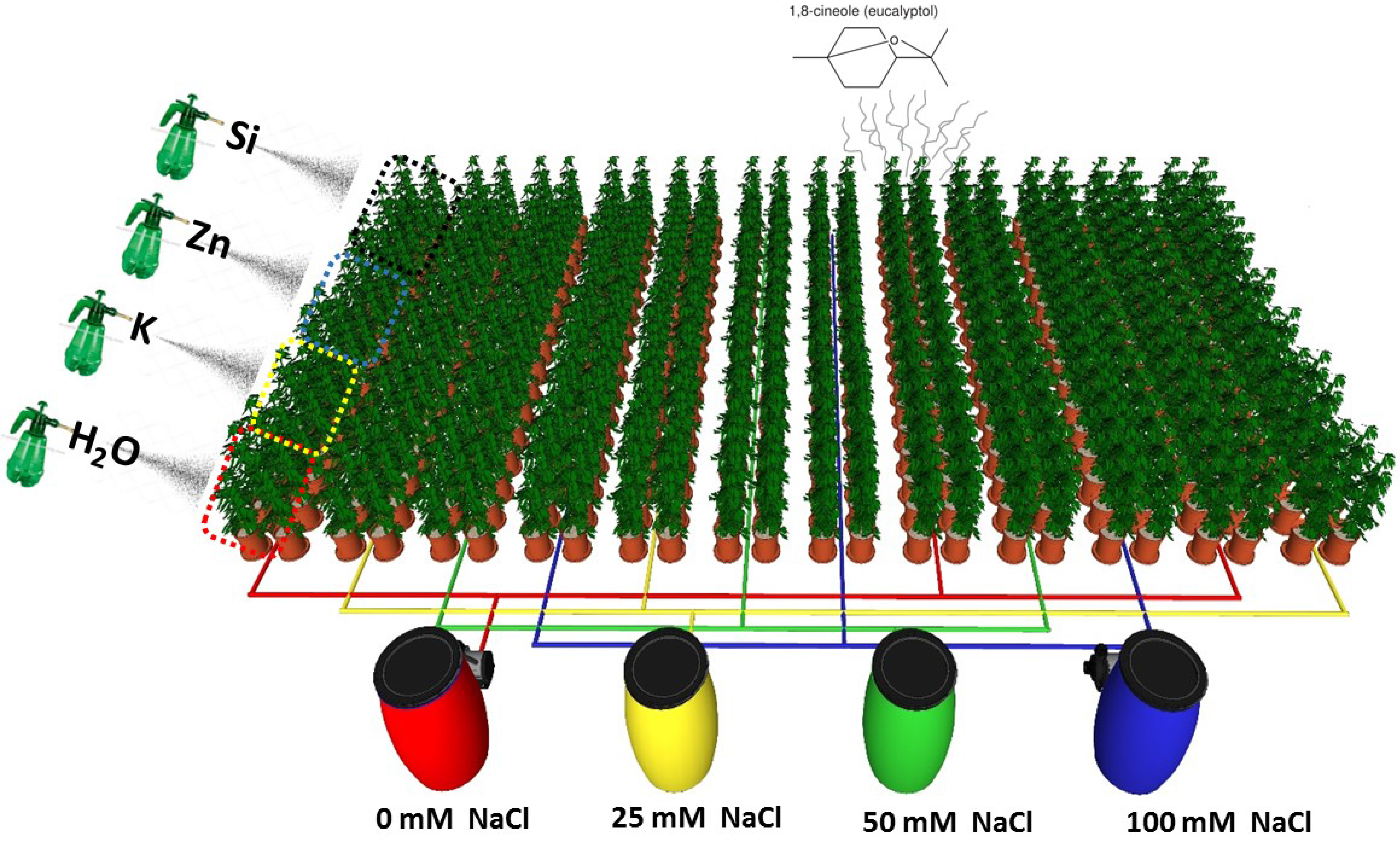


Supplementary Presentation 1. Experimental set up. Effects of salinity and/or cation foliar application in lavender grown hydroponically.
